# Identifying clinically useful biomarkers in neurodegenerative disease through a collaborative approach: the NeuroToolKit

**DOI:** 10.1186/s13195-023-01168-y

**Published:** 2023-01-28

**Authors:** Sterling C. Johnson, Marc Suárez-Calvet, Ivonne Suridjan, Carolina Minguillón, Juan Domingo Gispert, Erin Jonaitis, Agata Michna, Margherita Carboni, Tobias Bittner, Christina Rabe, Gwendlyn Kollmorgen, Henrik Zetterberg, Kaj Blennow

**Affiliations:** 1 University of Wisconsin School of Medicine and Public Health, University of Wisconsin-Madison, Madison, WI USA; 2 Geriatric Research Education and Clinical Center of the William S. Middleton Memorial Veterans Hospital, Madison, WI USA; 3 Barcelonaβeta Brain Research Center (BBRC), Pasqual Maragall Foundation, Wellington 30, 08005 Barcelona, Spain; 4 Centre for Biomedical Research Network on Frailty and Healthy Aging (CIBERFES), Instituto de Salud Carlos III, Madrid, Spain; 5 Neurology Service, Hospital del Mar, Barcelona, Spain; 6 IMIM (Hospital del Mar Medical Research Institute), Barcelona, Spain; 7 Roche Diagnostics International Ltd, Rotkreuz, Switzerland; 8 Centre for Biomedical Research in Network Bioengineering, Biomaterials and Nanomedicine, Instituto de Salud Carlos III, Madrid, Spain; 9 Roche Diagnostics GmbH, Penzberg, Germany; 10 F. Hoffmann-La Roche AG, Basel, Switzerland; 11 Genentech, A Member of the Roche Group, San Francisco, CA USA; 12 Department of Psychiatry and Neurochemistry, Institute of Neuroscience and Physiology, the Sahlgrenska Academy at the University of Gothenburg, Mölndal, Sweden; 13 Clinical Neurochemistry Laboratory, Sahlgrenska University Hospital, Mölndal, Sweden; 14 Department of Neurodegenerative Disease, UCL Institute of Neurology, Queen Square, London, UK; 15 UK Dementia Research Institute at UCL, London, UK; 16 Hong Kong Center for Neurodegenerative Diseases, Hong Kong, China

**Keywords:** Alzheimer’s disease, Amyloid-β, Cerebrospinal fluid biomarkers, Glial activation, Inflammation, Neurodegeneration

## Abstract

**Background:**

Alzheimer’s disease (AD) is a complex and heterogeneous disease, which requires reliable biomarkers for diagnosis and monitoring disease activity. Preanalytical protocol and technical variability associated with biomarker immunoassays makes comparability of biomarker data across multiple cohorts difficult. This study aimed to compare cerebrospinal fluid (CSF) biomarker results across independent cohorts, including participants spanning the AD *continuum*.

**Methods:**

Measured on the NeuroToolKit (NTK) prototype panel of immunoassays, 12 CSF biomarkers were evaluated from three cohorts (ALFA+, Wisconsin, and Abby/Blaze). A correction factor was applied to biomarkers found to be affected by preanalytical procedures (amyloid-β_1–42_, amyloid-β_1–40_, and alpha-synuclein), and results between cohorts for each disease stage were compared. The relationship between CSF biomarker concentration and cognitive scores was evaluated.

**Results:**

Biomarker distributions were comparable across cohorts following correction. Correlations of biomarker values were consistent across cohorts, regardless of disease stage. Disease stage differentiation was highest for neurofilament light (NfL), phosphorylated tau, and total tau, regardless of the cohort. Correlation between biomarker concentration and cognitive scores was comparable across cohorts, and strongest for NfL, chitinase-3-like protein-1 (YKL40), and glial fibrillary acidic protein.

**Discussion:**

The precision of the NTK enables merging of biomarker datasets, after correction for preanalytical confounders. Assessment of multiple cohorts is crucial to increase power in future studies into AD pathogenesis.

**Supplementary Information:**

The online version contains supplementary material available at 10.1186/s13195-023-01168-y.

## Introduction

High-quality, reliable, well-validated biomarkers, reflective of biological processes are required to understand the complexity of neurodegenerative diseases, such as Alzheimer’s disease (AD) [1]. In AD, using biomarkers would allow for increasing diagnostic accuracy, guiding patient stratification, monitoring the effect of treatment on underlying pathologies, and providing surrogate measures of disease activity to monitor and evaluate outcomes [2]. Comparability of cerebrospinal fluid (CSF) biomarkers between studies has been limited, partially due to methodology differences across cohorts. This issue has been somewhat circumvented by fully automated assays used in research and standardization of preanalytical procedures [3, 4]. Importantly, some CSF biomarker immunoassays (amyloid-β_1–42_ [Aβ42], phosphorylated 181P tau [pTau], and total tau [tTau]) are well validated for future wide-spread use in the clinical setting [5–7]. However, the challenge remains to have standardized clinical endpoints, statistical approaches, and immunoassay platforms that would enable unified biomarker utility and interpretation of results across independent multicenter studies.

The NeuroToolKit (NTK; Roche Diagnostics International Ltd) is a panel of 12 automated CSF immunoassays for biomarkers linked to neurodegeneration [8, 9]. This panel is designed to accelerate biomarker development in AD and other neurological disorders by generating robust, comparable, high-quality biomarker data across multiple research and clinical cohorts.

Using CSF biomarker data collected from participating sites, we aimed to address three prioritized research questions: (i) Comparative: Can a correction factor for biomarkers affected by different preanalytical procedures be applied that allows for comparison across multiple cohorts? (ii) Diagnostic: How much do the biomarker concentrations vary between cognitively unimpaired (CU) individuals and patients with mild cognitive impairment (MCI) or AD-dementia? (iii) Clinical: How well do biomarker concentrations correlate with clinical measures of cognition?

## Methods

This analysis utilizes data from three cohorts participating in the NTK project, which were selected to provide data spanning the entire AD *continuum*. The ALFA+ study (NCT02485730) aimed to characterize preclinical AD in CU individuals, most with a family history of AD (*n*=398) [8]. The Wisconsin cohort (*n*=651) comprised several longitudinal studies that utilized the same preanalytic protocol and included CU individuals, participants with MCI, or AD-dementia, enriched for parental history of AD [10]. The Abby/Blaze cohort (*n*=164) comprised participants in the ABBY (NCT01343966) and the BLAZE (NCT01397578) studies for patients with mild to moderate AD-dementia [11, 12]. Full eligibility criteria for each of the respective cohorts are described in the Supplementary Methods. All cohorts in the present analysis excluded participants who had comorbidities that would affect cognition. Some medications that affected cognition, such as sleep aids, were permitted in the Wisconsin cohort.

For the purposes of this analysis, the correction reference group for each cohort was defined as participants who were CU, *APOE-ε4* allele non-carriers, and aged <65 years. As the Abby/Blaze cohort only included participants with AD-dementia, a correction reference group could not be defined.

### Biomarkers

CSF biomarkers included chitinase-3-like protein-1 (YKL40), soluble triggering receptor expressed on myeloid cells 2 (sTREM2), glial fibrillary acidic protein (GFAP), interleukin (IL)-6, neurofilament light (NfL), neurogranin, S100, alpha-synuclein (α-Syn), amyloid-β_1–40_ (Aβ40), Aβ42, pTau, and tTau. CSF biomarker samples obtained at baseline/enrollment were included. All biomarkers were measured using the NTK panel of immunoassays, which currently includes the commercially available Elecsys β-amyloid (1–42) CSF, Elecsys total Tau CSF, and Elecsys phospho-Tau (181P) CSF immunoassays, and robust prototype assays for the nine remaining biomarkers. Biomarkers Aβ42, Aβ40, pTau, tTau, s100, and IL-6 were measured using the cobas e 601 analyzer, and the remaining biomarkers were measured using the cobas e 411 analyzer (both Roche Diagnostics International Ltd).

### Preanalytical factor correction

The preanalytical procedures employed by each cohort are detailed in the Supplementary Materials. Sample collection within the Wisconsin cohort was initiated ahead of standardized preanalytical protocol dissemination [9]; therefore, the correction factors are calculated in the respective correction reference groups (participants who were CU, *APOE*-ε4 allele non-carriers, and aged <65 years) of the Wisconsin and ALFA+ cohorts assuming the ALFA+ cohort being the “standard cohort.” The correction factor was calculated using the formula:

$$\textrm{Correction}\ \textrm{factor}=\textrm{median}\left(\textrm{ALFA}+\textrm{cohort}\right)/\textrm{median}\left(\textrm{Wisconsin}\ \textrm{cohort}\right)$$

Application of the correction factor was deemed successful in accounting for preanalytical variations by assessment of biomarker distribution overlap before and after correction, i.e., if following correction the biomarker distributions had good overlap, the correction was a success. The correction was applied to CSF biomarkers: α-Syn, Aβ40, and Aβ42 (Table S1), which are known to be significantly affected by preanalytical protocols, specifically related to the ability of these biomarkers to stick to the tubes used during testing [4, 13]. Conversely, the remaining biomarkers, such as pTau and tTau, appear to be unaffected by the tubes employed [4]. Natural variations between the cohorts were unaffected. Variations between cohorts may also result from inherent cohort differences or from cultural bias, e.g., in cognitive assessments.

### CSF amyloid-β cut-off value derivation

Amyloid-β pathology was determined by CSF Aβ42/Aβ40 ratio for this analysis; the results are provided in the Supplementary Materials. To derive the cut-off values for the CSF Aβ42/Aβ40 ratio, Gaussian mixture modeling was independently applied to the ALFA+ and Wisconsin cohorts. The optimal number of Gaussians was set as two, after testing models with two, three, and four Gaussians (Supplementary Materials). Derived cut-off values were defined as *x*±2*s with differently defined parameters for *x* and *s*: (i) *x*=*μ*, *s*=*σ* (mu, sigma; Gaussian parameters of the amyloid-β negative [A−] population); (ii) *x*=mean, *s*=SD of samples assigned to A− population; and (iii) *x*=median, *s*=rSD of samples assigned to A− population (Tables S2–S5). The resulting cut-off values for the Aβ42/Aβ40 ratio were defined as 0.071 for the ALFA+ cohort [8] and 0.060 (0.075 after correction) for the Wisconsin cohort. Only patients with AD-dementia were included in the ABBY and BLAZE studies; therefore, cut-off values were not defined as this cohort was not divided by amyloid-β status. For comparison, the cut-off values determined for the ALFA+ cohort were applied to the Wisconsin cohort after correction.

### Cognitive assessments

All participants completed the MMSE [14] and Clinical Dementia Rating scale Sum of Boxes (CDR-SB) [15] cognitive assessments during the respective studies. The time between CSF biomarker collection at baseline/enrollment and cognitive assessment varied for each participant and in some cases was up to 1 year. For the longitudinal studies, the cognitive assessment closest to the first lumbar puncture was used in this analysis, including those cognitive assessments performed before the lumbar puncture. The ALFA+ cohort only included participants with CDR-SB=0, per the study exclusion criteria [16]. Calculations of a modified Preclinical Alzheimer Cognitive Composite (PACC) were based on methods proposed by Donohue et al. [17], Papp et al. [18], and Jonaitis et al. [19]. Variables included in the composite in the ALFA+ cohort were Semantic Fluency (animal naming), Free and Cued Selective Reminding Test with Total Immediate Recall, and Wechsler Adult Intelligence Scale-Revised Coding subtest. In the Wisconsin cohort, Semantic Fluency (animal naming), Rey Auditory Verbal Learning Test Trials 1–5 Sum, and Wechsler Adult Intelligence Scale-Revised Coding subtest were included. PACC was not used for the Abby/Blaze cohort.

### Statistical analyses

To compare biomarker concentrations across cohorts, the median concentration and interquartile range of all NTK CSF biomarkers before and after correction were calculated for all cohorts, therefore enabling the inclusion of outlying samples. The robust-to-outliers standard deviation (rSD) was estimated based on percentile values (rSD=[value of 84.13% percentile − value of 15.87% percentile]/2). The distributions of the CSF biomarker concentrations within the same disease stage across the cohorts were statistically compared before and after correction. To compare baseline/enrollment values for each CSF biomarker for both CU individuals and patients with AD-dementia separately, correlation values were computed using Spearman’s rho.

Fold change was calculated using the canonical fold change calculation in CSF biomarker concentrations in CU A− individuals from either the ALFA+ or Wisconsin cohorts with (i) CU amyloid-β-positive (A+) individuals (ALFA+), (ii) CU A+ individuals (Wisconsin), (iii) patients with MCI A+ (Wisconsin), (iv) patients with AD-dementia (Wisconsin), and (v) patients with AD-dementia (Abby/Blaze). Receiver operating characteristic (ROC) analyses, presented with an area under the curve (AUC) and 95% confidence intervals, comparing CSF biomarker concentrations in CU A− individuals with (i) CU A+ individuals (ALFA+), (ii) CU A+ individuals (Wisconsin), (iii) patients with MCI A+ (Wisconsin), and (iv) and patients with AD-dementia (Wisconsin) were performed.

Spearman’s rho correlation between the concentration of all the biomarkers and cognitive performance, reflected in MMSE and/or PACC scores, in the different disease stages was computed. To assess cognitive scores from each cohort on a similar scale relative to that cohort’s control participants, standardization of each individual raw score into *z*-scores was performed using the means and rSDs obtained from the A− samples of each control group as a reference. All three obtained *z*-scores were averaged. The obtained PACC values were re-standardized using the mean and rSD from the A− samples of the control group. Missing data in any of the raw scores led to a missing PACC value.

## Results

**Fig. 1:**
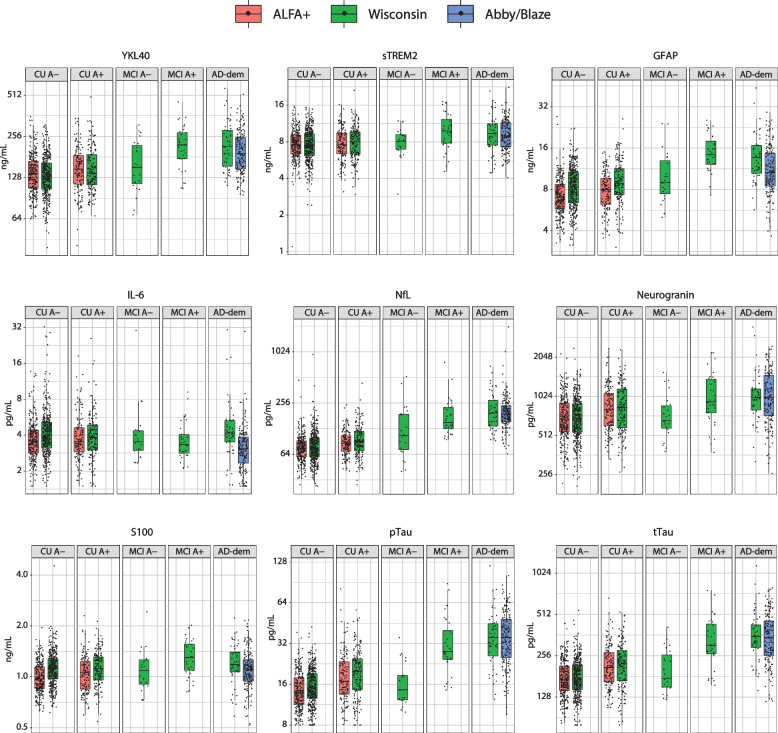
Box plots of uncorrected biomarkers by disease stage and amyloid-β status as defined by Aβ42/Aβ40 ratio. Aβ40, amyloid-β_1–40_; Aβ42, amyloid-β_1–42_; AD, Alzheimer’s disease; CU, cognitively unimpaired; GFAP, glial fibrillary acidic protein; MCI, mild cognitive impairment; NfL, neurofilament light; pTau, phosphorylated tau; tTau, total tau; YKL40, chitinase-3-like protein-1

**Fig. 2:**
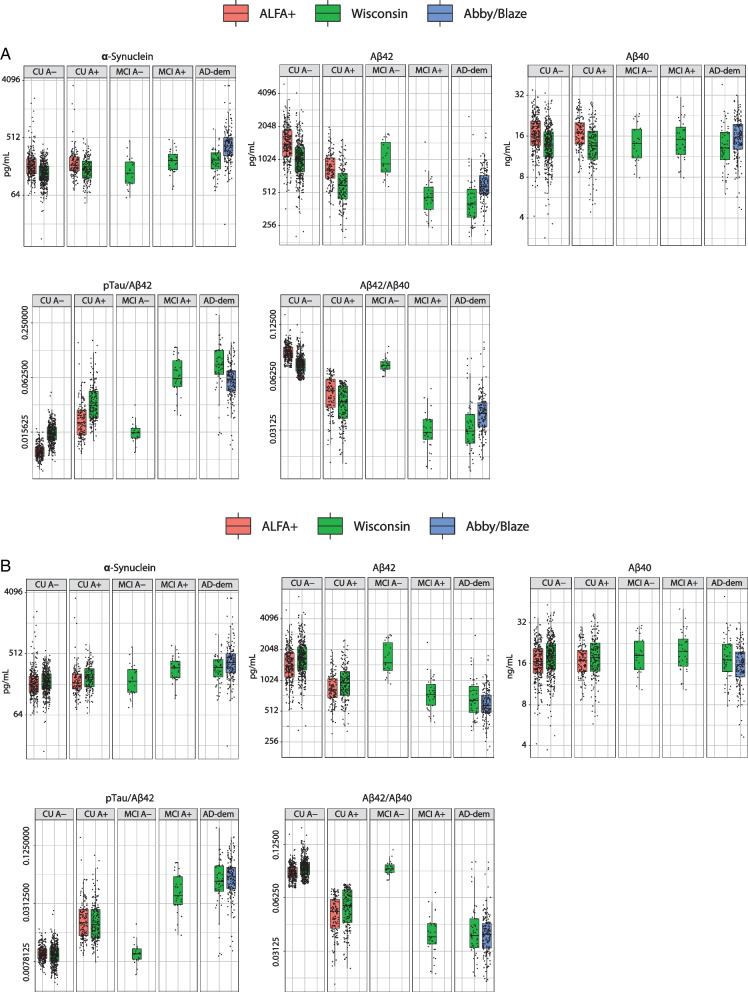
Box plots of biomarkers by disease stage and amyloid-β status as defined by Aβ42/Aβ40 ratio; **A** uncorrected values and **B** corrected values. Aβ40, amyloid-β_1–40_; Aβ42, amyloid-β_1–42_; AD, Alzheimer’s disease; CU, cognitively unimpaired; MCI, mild cognitive impairment; pTau, phosphorylated tau

No differences were found between cohorts in age, sex, years of education, MMSE score, or *APOE-ε4* carriership status within the different disease stages (Table 1). Prior to correction for preanalytical protocol differences, concentrations of α-Syn, Aβ40, and Aβ42 were significantly higher in CU A− individuals in the ALFA+ cohort compared with the Wisconsin cohort. Biomarker distributions for the ALFA+ and Wisconsin cohorts (Fig. S1) illustrate that biomarker concentrations were comparable between cohorts in the reference groups following correction. CSF biomarkers GFAP, IL-6, S100, pTau, and pTau/Aβ42 were significantly lower in the ALFA+ cohort compared with the Wisconsin cohort in CU A− individuals (Table 2). These differences may arise from intrinsic cohort differences or from measurement bias; therefore, the raw values of these biomarkers cannot be directly compared between cohorts, but the trends within cohorts can be compared with each other. For the remaining biomarkers, there were no significant differences between cohorts. The details of all the CSF biomarker concentrations, including before and after correction for those affected, across all cohorts and disease stages are reported in Table 2. Biomarker concentration distributions before and after correction represented as boxplots are shown in Figs. 1 and 2. Classification of amyloid-β status, including the use of a single cut-off value for both the ALFA+ and Wisconsin cohorts, had no impact on the comparability of biomarker concentrations across cohorts (Table S6, Table S7).

**Table 1:** Characterization of cohorts (amyloid status as defined by Aβ42/Aβ40 ratio)

	CU (A−)		CU (A+)		CU (missing)^a^	MCI (A−)	MCI (A+)	AD-dementia (all)	
Characteristics	ALFA+	Wisconsin	ALFA+	Wisconsin	Wisconsin	Wisconsin	Wisconsin	Wisconsin	Abby/Blaze
*N*	263	376	135	160	10	23	33	49	164
Age, mean (SD)	60.5 (4.5)	60.3 (7.5)	62.2 (5.0)	64.0 (7.2)	60.7 (7.6)	69.2 (8.5)	73.7 (7.9)	72.3 (8.5)	69.6 (7.8)
Female, *n* (%)	163 (41)	250 (38)	81 (20)	107 (16)	8 (1)	12 (2)	12 (2)	17 (3)	85 (52)
Education, mean (SD)	13.6 (3.5)	16.2 (2.5)	13.3 (3.6)	16.3 (2.4)	17.1 (3.1)	15.7 (2.7)	16.2 (2.6)	14.5 (2.7)	NA
MMSE, mean (SD)	29.2 (0.9)	29.4 (0.9)	29.1 (1.0)	29.2 (1.0)	29.3 (1.0)	28.2 (1.7)	27.0 (2.2)	21.7 (3.8)	21.7 (3.2)
CDR-SB, mean (SD)	0	0.04 (0.20)	0	0.13 (0.31)	0	1.38 (1.44)	1.81 (1.18)	4.44 (1.61)	4.56 (1.94)
PACC, mean (SD)	−0.16 (0.93)	−0.13 (1.13)	−0.11 (1.03)	−0.35 (1.29)	−0.02 (0.98)	−2.26 (1.04)	−2.93 (0.97)	−4.60 (1.42)	NA
*APOE-ε4* carriers, *n* (%)	111 (28)	105 (16)	103 (26)	86 (13)	4 (1)	6 (1)	20 (3)	32 (5)	118 (72)
*APOE-ε4* non-carriers, *n* (%)	152 (38)	251 (39)	32 (8)	67 (10)	6 (1)	13 (2)	12 (2)	16 (3)	46 (28)

^a^Amyloid-β status is unknown due to missing Aβ40 analysis

*Abbreviations*: *Aβ42*, amyloid-β_1–42_; *Aβ40*, amyloid-β_1–40_; *AD*, Alzheimer’s disease; *CDR-SB*, Clinical Dementia Rating Scale – Sum of Boxes; *CU*, cognitively unimpaired; *MCI*, mild cognitive impairment; *MMSE*, Mini-Mental State Examination; *NA*, not applicable; *PACC*, Preclinical Alzheimer’s Cognitive Composite; *SD*, standard deviation

**Table 2:** Biomarker data for each cohort, including uncorrected and corrected data (amyloid status as defined by Aβ42/Aβ40 ratio)

	CU (A−)			CU (A+)			CU (missing)^a^	MCI (A−)	MCI (A+)	AD-dementia (all)		
Characteristics	ALFA+	Wisconsin	***p***	ALFA+	Wisconsin	***p***	Wisconsin	Wisconsin	Wisconsin	Wisconsin	Abby/Blaze	***p***
*N*	263	376		135	160		10	23	33	49	164	
**Uncorrected CSF biomarkers, median (IQR)**												
YKL40, ng/mL	136.2 (60.6)	131.8 (58.2)	0.173	147.6 (70.6)	138.0 (74.9)	0.548	154.8 (64.1)	152.1 (104.5)	222.0 (96.8)	216.4 (129.2)	191.6 (103.9)	0.112
sTREM2, ng/mL	7.57 (2.74)	7.51 (3.15)	0.163	7.58 (3.05)	7.96 (3.33)	0.875	9.07 (2.59)	8.11 (2.24)	9.81 (4.39)	9.36 (3.74)	8.93 (4.15)	0.844
GFAP, ng/mL	7.00 (2.93)	8.34 (4.30)	<0.001	8.02 (3.43)	8.84 (3.98)	0.002	8.72 (3.75)	9.02 (5.54)	14.33 (5.72)	13.62 (6.24)	10.58 (6.27)	0.002
IL-6, pg/mL	3.57 (1.58)	3.97 (2.01)	<0.001	3.52 (1.71)	3.83 (1.84)	0.469	NA	3.52 (1.38)	3.33 (1.21)	4.17 (1.78)	3.08 (1.52)	<0.001
NfL, pg/mL	75.6 (31.9)	75.8 (39.5)	0.481	86.2 (36.0)	90.8 (48.8)	0.222	66.6 (34.7)	106.6 (116.2)	151.0 (98.8)	195.2 (137.4)	189.6 (82.6)	0.908
Neurogranin, pg/mL	683.7 (360.7)	693.4 (353.0)	0.162	805.9 (466.4)	843.7 (574.5)	0.914	805.7 (439.3)	666.8 (285.4)	928.1 (620.2)	1006.0 (362.9)	999.9 (757.9)	0.908
S100, ng/mL	0.97 (0.29)	1.12 (0.31)	<0.001	1.06 (0.38)	1.12 (0.35)	0.010	0.92 (0.23)	1.09 (0.35)	1.30 (0.47)	1.18 (0.33)	1.10 (0.32)	0.038
α-Syn, pg/mL	179.4 (99.6)	141.7 (74.7)	<0.001	190.5 (101.7)	162.6 (100.6)	<0.001	169.6 (71.3)	142.7 (115.1)	226.1 (121.0)	229.2 (134.3)	379.5 (245.6)	<0.001
Aβ40, ng/mL	16.6 (7.0)	14.0 (6.0)	<0.001	16.9 (6.0)	13.6 (6.6)	<0.001	NA	14.1 (7.0)	15.1 (6.9)	13.1 (6.4)	15.8 (6.7)	0.007
Aβ42, pg/mL	1417.0 (779.5)	1029.0 (530.3)	<0.001	821.6 (366.0)	592.9 (315.0)	<0.001	431.1 (231.1)	934.9 (689.7)	459.9 (214.3)	401.9 (243.3)	576.8 (234.5)	<0.001
pTau, pg/mL	13.8 (6.6)	15.1 (6.5)	0.013	16.8 (10.0)	20.0 (10.1)	0.129	8.0 (0.0)	14.6 (6.3)	27.9 (15.4)	35.6 (18.9)	35.5 (23.0)	0.908
tTau, pg/mL	171.0 (71.1)	173.1 (74.3)	0.539	209.5 (105.4)	223.1 (111.4)	0.639	108.1 (28.1)	175.1 (108.4)	304.9 (171.5)	351.7 (135.9)	345.6 (201.9)	0.908
pTau/Aβ42	0.0093 (0.0022)	0.0150 (0.0049)	<0.001	0.0199 (0.0127)	0.0306 (0.0218)	<0.001	0.0400 (0.0000)	0.0153 (0.0038)	0.0612 (0.0466)	0.0860 (0.0565)	0.0590 (0.0293)	<0.001
Aβ42/Aβ40	0.0857 (0.0123)	0.0737 (0.0120)	<0.001	0.0523 (0.0188)	0.0455 (0.0192)	<0.001	NA	0.0730 (0.0073)	0.0303 (0.0083)	0.0308 (0.0120)	0.0389 (0.0121)	<0.001
**Corrected CSF biomarkers, median (IQR)**												
α-Syn, pg/mL	179.4 (99.6)	199.3 (105.0)	0.173	190.5 (101.7)	228.7 (141.4)	0.025	238.5 (100.2)	200.7 (161.9)	318.0 (170.2)	322.3 (188.9)	379.5 (245.6)	0.109
Aβ40, ng/mL	16.6 (7.0)	18.3 (7.8)	0.094	16.9 (6.0)	17.8 (8.6)	0.273	NA	18.5 (9.1)	19.7 (9.0)	17.1 (8.3)	15.8 (6.7)	0.250
Aβ42, pg/mL	1417.0 (779.5)	1670.2 (860.7)	0.001	821.6 (366.0)	962.3 (511.3)	0.027	699.7 (375.1)	1517.4 (1119.5)	746.5 (347.8)	652.3 (394.9)	576.8 (234.5)	0.250
pTau/Aβ42	0.0093 (0.0022)	0.0093 (0.0030)	0.173	0.0199 (0.0127)	0.0188 (0.0135)	0.453	0.0246 (0.0000)	0.0094 (0.0023)	0.0377 (0.0287)	0.0530 (0.0348)	0.0590 (0.0293)	0.256
Aβ42/Aβ40	0.0857 (0.0123)	0.0916 (0.0149)	<0.001	0.0523 (0.0188)	0.0566 (0.0239)	0.008	NA	0.0907 (0.0091)	0.0377 (0.0103)	0.0383 (0.0149)	0.0389 (0.0121)	0.908

*p*-values are adjusted using the FDR method for each cohort, amyloid-β status, and disease stage comparison independently

*Abbreviations*: *Aβ42*, amyloid-β_1–42_; *Aβ40*, amyloid-β_1–40_; *α-Syn*, alpha-synuclein; *AD*, Alzheimer’s disease; *CU*, cognitively unimpaired; *CSF*, cerebrospinal fluid; *FDR*, false discovery rate; *GFAP*, glial fibrillary acidic protein; *IL*, interleukin; *IQR*, interquartile range; *MCI*, mild cognitive impairment; *NA*, not applicable; *NfL*, neurofilament light; *pTau*, phosphorylated tau; *sTREM2*, soluble triggering receptor expressed on myeloid cells 2; *tTau*, total tau; *YKL40*, chitinase-3-like protein-1

^a^Amyloid-β status unknown due to missing Aβ40 analysis

Of the biomarker values that did not undergo correction, YKL40, GFAP, NfL, neurogranin, pTau, and tTau were all increased in patients with AD-dementia or MCI who were A+ compared with CU individuals and patients with MCI who were A−. Both α-Syn and the pTau/Aβ42 ratio values were increased in patients with AD-dementia or MCI who were A+. Values for Aβ42 and the Aβ42/Aβ40 ratio were decreased in patients with AD-dementia or MCI. These results applied to both the corrected and uncorrected values. Correlations of CSF biomarkers were comparable across cohorts within the same disease stage (Supplementary Results, Fig. S2).

### Diagnostic variations

**Fig. 3:**
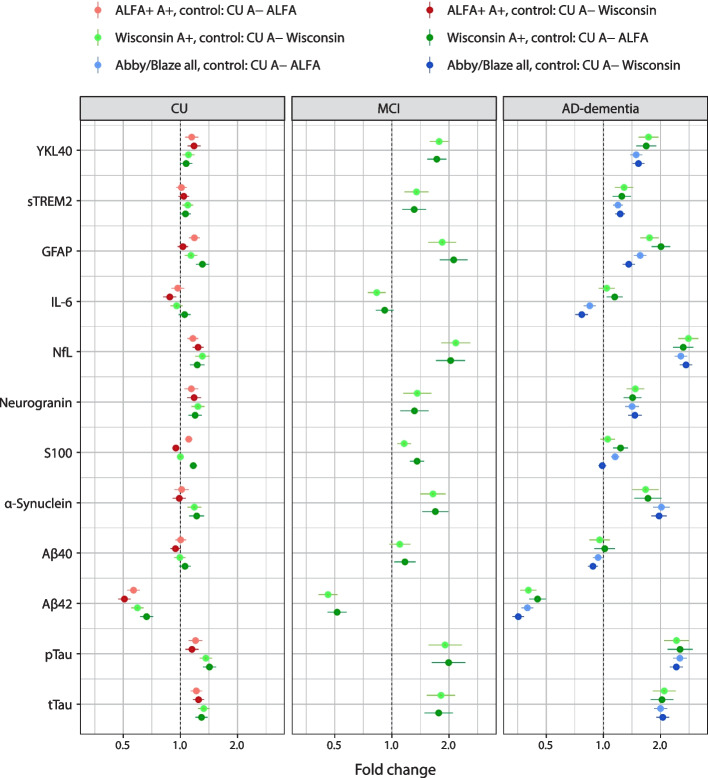
Fold change vs CU, Aβ42/Aβ40 amyloid-β negative with age <65 years. Aβ40, amyloid-β_1–40_; Aβ42, amyloid-β_1–42_; AD, Alzheimer’s disease; CU, cognitively unimpaired; GFAP, glial fibrillary acidic protein; IL, interleukin; MCI, mild cognitive impairment; NfL, neurofilament light; pTau, phosphorylated tau; sTREM2, soluble triggering receptor expressed on myeloid cells 2; tTau, total tau; YKL40, chitinase-3-like protein-1

The fold change in CSF biomarker concentration compared with CU A− individuals was comparable across cohorts (Tables S8–S10). Across all cohorts, the fold change showed a similar pattern regardless of whether it was calculated using values from the CU A− individuals group from the same cohort or from a different cohort. NfL, pTau, and tTau concentrations were higher in patients with AD-dementia, followed by patients with MCI, compared with CU individuals. Figure 3 shows the fold change displayed as a forest plot of the biomarker concentration compared with the CU A− individual group (derived from either the Wisconsin cohort or the ALFA+ cohort).

**Fig. 4:**
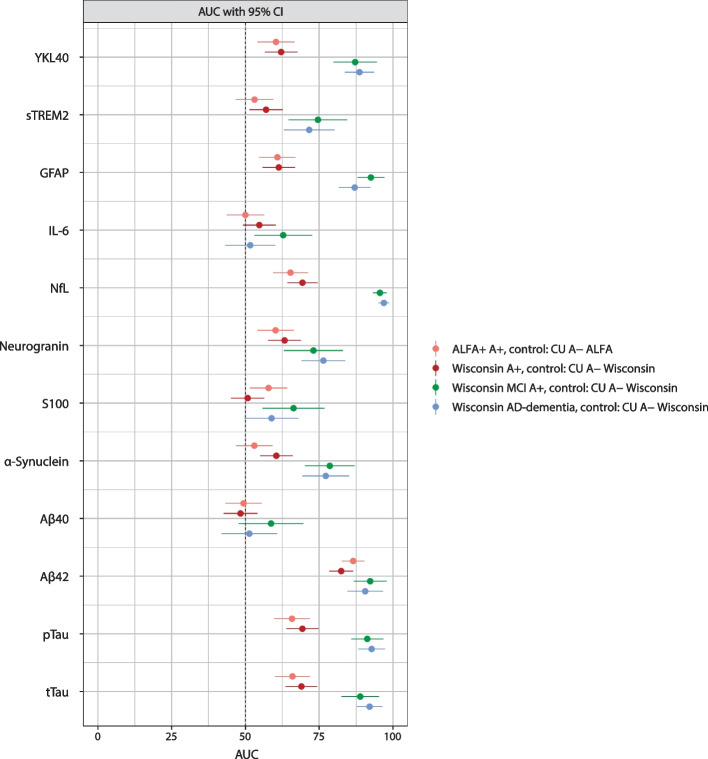
Forest plot of ROC analyses AUC with 95% confidence intervals (amyloid status as defined by Aβ42/Aβ40 ratio). Aβ40, amyloid-β_1–40_; Aβ42, amyloid-β_1–42_; AD, Alzheimer’s disease; AUC, area under the curve; CI, confidence interval; CU, cognitively unimpaired; GFAP, glial fibrillary acidic protein; IL, interleukin; MCI, mild cognitive impairment; NfL, neurofilament light; pTau, phosphorylated tau; ROC, receiver operating characteristics; sTREM2, soluble triggering receptor expressed on myeloid cells 2; tTau, total tau; YKL40, chitinase-3-like protein-1

ROC analyses (Fig. S3) and AUC data (Fig. 4 and Table S11) confirmed the results shown by fold change. Among the CSF biomarkers evaluated, NfL (followed by YKL40, GFAP, Aβ42, pTau, and tTau) had the greatest diagnostic value for discriminating CU A− individuals from patients with MCI or AD-dementia. The results were not affected by amyloid-β status as Aβ42 showed the greatest ability of all the biomarkers measured to discriminate CU A− individuals from CU A+ individuals in both the ALFA+ and Wisconsin cohorts.

### Correlation with clinical measures of cognition

**Fig. 5:**
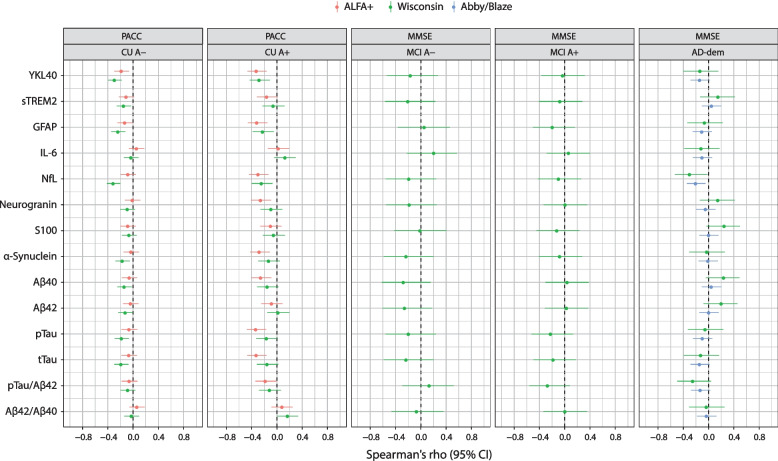
Baseline correlation between NTK biomarkers and cognitive scores (amyloid status as defined by Aβ42/Aβ40 ratio). Aβ40, amyloid-β_1–40_; Aβ42, amyloid-β_1–42_; AD, Alzheimer’s disease; CI, confidence interval; CU, cognitively unimpaired; GFAP, glial fibrillary acidic protein; IL, interleukin; MCI, mild cognitive impairment; NfL, neurofilament light; NTK, NeuroToolKit; PACC, Preclinical Alzheimer Cognitive Composite; pTau, phosphorylated tau; sTREM2, soluble triggering receptor expressed on myeloid cells 2; tTau, total tau; YKL40, chitinase-3-like protein-1

The correlation between CSF biomarker concentration and cognitive scores (MMSE and/or PACC) appears comparable across cohorts. The correlation of the biomarker concentration and the cognitive scores in the different disease stages and cohorts is shown as a forest plot in Fig. 5. For CU A+ individuals, the strongest correlation among different biomarker concentrations and PACC score was found in YKL40, GFAP, and NfL. For patients with AD-dementia (Wisconsin and Abby/Blaze cohorts), the strongest correlation between biomarker concentrations and MMSE was found in NfL.

## Discussion

The NTK project involves the evaluation of a large panel of CSF biomarkers of AD pathology and glial activity that may address the goals outlined in the introduction for diagnosis, prognosis, predicting treatment response, and surrogate outcomes in AD. Following recently published studies describing the utility of the NTK immunoassays in clinical settings [8, 9, 20], this analysis demonstrates that the application of a correction factor for biomarkers affected by preanalytical variability improves the comparability of NTK data across three independent cohorts spanning the AD *continuum*. After establishing the adequacy of the correction factor, we examined the cohorts for insights regarding biomarker relationships across the disease stages. In response to the three prioritized research questions discussed in the introduction, this analysis provided the following answers.

Comparative: The correction factor for preanalytical protocol variations employed here enabled the comparison of data across cohorts, rendering significant differences in CSF biomarker concentrations irrelevant. The correction factor was not developed specifically for the present cohorts and is generalizable, which enables the introduction of further cohorts into the comparative framework. Comparability of CSF biomarker data acquired with the NTK was found to be robust to methods of amyloid-β status classification, suggesting the possibility of introducing future cohorts with or without having calculated their own cut-off values. Further investigation with additional cohorts is needed. Correlations between all CSF biomarkers were consistent for CU individuals in both the ALFA+ and Wisconsin cohorts and for patients with AD-dementia in both the Wisconsin and Abby/Blaze cohorts. These results indicate that the data produced by the NTK were comparable across cohorts, meaning results and subsequent analyses may be combined.

Diagnostic: CSF biomarker differences for CU A− individuals compared with patients with AD-dementia, were comparable across cohorts, regardless of the CU A− individual cohort origin. The fold change for CU A− individuals compared with patients with MCI and patients with AD-dementia was largest for NfL, pTau, and tTau. The diagnostic utility of the aforementioned biomarkers was confirmed with ROC and AUC analyses, suggesting these biomarkers, and Aβ42, are the most appropriate for disease stage differentiation.

Clinical: Correlations of CSF biomarker concentrations with cognitive score were comparable across cohorts. Associations between biomarker concentrations and clinical cognitive scores have been the focus of several studies to validate measures of cognition but only few studies include a panel of CSF biomarkers [21–25]. Correlations between CSF biomarker concentrations and cognitive scores are indicative of the prognostic utility of the biomarker in question [21]. Here, we found that clinical cognitive scores had the strongest correlation with NfL, in the CU A+ and AD-dementia disease stages. For CU A− individuals, NfL correlated with the PACC score in the Wisconsin cohort, but less so in the ALFA+ cohort. Our findings on the moderate, inverse correlation of NfL with clinical measures of cognition are consistent with the literature [26] indicating markers of neurodegeneration track with cognition. No strong correlations of biomarkers for patients with MCI A−/A+ were observed.

Consistently across the three prioritized research questions examining correlations with biomarker concentrations, NfL is the most promising CSF biomarker for AD along with the well-established Aβ42, pTau, and tTau. No statistically significant differences in NfL concentration between cohorts were observed within disease stage. Additionally, NfL differentiated disease stage and correlated with measures of cognition. The observation of NfL differentiation between disease stages is consistent with recent literature, which includes the correlation of NfL with imaging markers of neurodegeneration [27]. However, NfL is not a specific biomarker for AD, but rather for several neuroinflammatory diseases [28–30]. Analyzing the biomarker results of NfL within the context of other biomarkers of neuroinflammation, glial activity, and known AD pathology is key to the utility of NfL within the AD landscape. As such, the NTK panel provides clinical utility from both the biomarkers at an individual level and as a whole. In the current study, NfL did not strongly correlate with the other biomarkers included in the NTK; these results are in contrast to the literature, which describes NfL correlations with (plasma) GFAP [31], pTau, tTau, and neurogranin [26].

In the AD field, vast amounts of biomarker data are generated; however, comparability between biomarker datasets is not routinely employed due to the heterogeneity of the disease and the technical variability associated with preanalytical protocols and the immunoassay platforms used. Several pre-competitive efforts to generate comparable datasets, harmonize and maximize the interpretability of the biomarker results collected, such as the NTK project and the Global Biomarker Standardization Consortium (GBSC; a collaborative effort in the acceleration of biomarker standardization [32]), are ongoing. Measures have also been taken to standardize methods of CSF collection and measurement to ensure reproducible and consistent results across multiple cohorts and immunoassays [3, 4]. The aim is to clarify the clinical utility of biomarkers to inform clinical trial design and for diagnostic development.

To our knowledge, the NTK project is the first large-scale project that aims to generate robust and comparable biomarker data across multiple independent cohorts in AD. While cross-cohort examinations of datasets have been employed for single biomarker validation studies [33], a project with the number of biomarkers described in this analysis is uncommon. Other large-scale studies investigating the utility of biomarkers at various disease stages include the Alzheimer’s Disease Neuroimaging Initiative (ADNI) [34] and the Australian Imaging, Biomarkers and Lifestyle (AIBL) study of aging [35]. However, these studies do not include multiple cohorts and are geographically restricted, and the biomarkers are measured using multiple immunoassay platforms. The NTK project seeks to maximize the comparability of biomarker measurements between cohorts by generating and analyzing biomarker data using a common platform and immunoassay.

A key strength of this study is the large dataset included, spanning several countries and native languages. Importantly, the cloud-based DRE employed in the NTK project allows for an increase in data sharing with interpretation unified across several cohorts. The researchers involved in the NTK project retain control over their data and through collaborative efforts they can work with other consenting cohorts to enrich their datasets and provide further insights. The standardized statistical analysis on single cohorts, as well as the correction procedure, comparisons, and graphical representations across different cohorts described here, can also be applied to other cohorts via the NTK app (currently under development). Following the approval and willingness of collaboration of all data owners, and with the accrual of added data and cohorts, the feasibility of the NTK app will be thoroughly explored.

## Limitations

Possible limitations of the present study include the decision not to use amyloid-β positron emission tomography data as a common comparator across cohorts to determine amyloid-β status, and the use of only single-timepoint data for individual assessments (e.g., only one MMSE assessment was included per individual). Future studies should include the clinical follow-up data for the participants. In the longitudinal cohorts included in this study, CSF sample collection and cognitive assessments were not necessarily completed at the same time. Cognitive assessments and CSF sample collection were performed within a year of each other, or the sample was excluded from this analysis. Although AD is a slowly evolving disease, this may have led to variations in the correlations for these measures. However, for individuals with MCI and CU individuals, pTau, tTau, Aβ42, and neurogranin have been shown to be stable over a 2-year period [36, 37]. In addition, the Abby/Blaze cohort of individuals with AD-dementia, cognitive assessment, and CSF sample collection both occurred at baseline. Compared with the CU (*n*=944) and AD-dementia (*n*=213) patient populations, the MCI population (*n*=56) is relatively small. Expanding the results within this important patient population may lead to further insights into AD disease pathogenesis and the clinical utility of the NTK panel of immunoassays.

The correction for preanalytical protocols was based on several assumptions: (i) the ALFA+ cohort samples were all collected according to the standardized protocol, but the Wisconsin cohort samples were not; (ii) sample handling procedures only affect α-Syn, Aβ42, and Aβ40, which may be disproved with future research; (iii) sample handling procedures have a similar effect on samples with high concentrations and those with lower concentrations. These assumptions may explain for the variations between cohorts seen in the biomarker values that were not corrected; however, it is beyond the scope of this study to determine the cause of such variations. In addition, correction for preanalytical protocols was employed and not harmonization of all biomarker results. While harmonization of the biomarker results would have allowed for head-to-head analysis, as no bridging samples (measured on the same instrument at the same time from all cohorts) required to perform a harmonization were collected correction was employed. The rationale for the correction factor was that existing cohorts were used, meaning some of the samples had been collected prior to the initiation of the present analysis. As such, reference materials and methods were not used. The correction factor is therefore employed as a solution to enable the inclusion of these data. If the analysis were to be performed as part of a prospective study, the correction factor may not be required if standardized procedures and reference materials were employed.

In summary, the robust prototype NTK panel of immunoassays provides biomarker data that can be used to support the utility of biomarkers in clinical trials and in the diagnostic clinical setting. Our study supports the feasibility of cross-cohort collation of data provided by the NTK immunoassays to enable further insight gathering into the underlying pathogenesis of AD. Our next step is to use a DRE to implement the standardized statistical analysis plan and increase the interpretation of results across studies. In addition, the NTK project will expand to include supplementary CSF immunoassays beyond the 12 in this study, as well as plasma biomarker immunoassays.

## Supplementary Information

**Additional file 1.** Supplementary methods, tables, and figures.

## Data Availability

ALFA+ cohort: All requests for raw and analyzed data and materials will be promptly reviewed by the senior authors to verify whether the request is subject to any intellectual property or confidentiality obligations. Bulk anonymized data can be shared by request from any qualified investigator for the sole purpose of replicating procedures and results presented in the article, providing data transfer is in agreement with European Union legislation and decisions by the institutional review board of each participating center. Wisconsin cohort: Deidentified data from the Wisconsin cohort are available by request through our website. All requests will be reviewed by study leadership, and if approved, data will be transferred subject to an institutional data use agreement. Abby/Blaze cohort: Qualified researchers may request access to individual anonymised patient-level data through the clinical study data request platform (https://vivli.org/). Further details on Roche’s criteria for eligible studies are available here: https://vivli.org/members/ourmembers/. For further details on Roche’s Global Policy on the Sharing of Clinical Information and how to request access to related clinical study documents, see here: https://www.roche.com/research_and_development/who_we_are_how_we_work/clinical_trials/our_commitment_to_data_sharing.htm.

## Abbreviations

α-Syn: Alpha-synuclein
Aβ40: Amyloid-β_1–40_
Aβ42: Amyloid-β_1–42_
AD: Alzheimer’s disease
ADNI: Alzheimer’s Disease Neuroimaging Initiative
AIBL: Australian Imaging, Biomarkers and Lifestyle
AUC: Area under the curve
CDR-SB: Clinical Dementia Rating scale Sum of Boxes
CSF: Cerebrospinal fluid
CU: Cognitively unimpaired
DRE: Digital Research Environment
GBSC: Global Biomarker Standardization Consortium
GFAP: Glial fibrillary acidic protein
IL: Interleukin
IVD: In vitro diagnostic
MCI: Mild cognitive impairment
MMSE: Mini-Mental State Examination
NfL: Neurofilament light
NTK: NeuroToolKit
PACC: Preclinical Alzheimer Cognitive Composite
pTau: Phosphorylated tau
ROC: Receiver operating characteristic
rSD: Robust-to-outliers standard deviation
sTREM2: Soluble triggering receptor expressed on myeloid cells 2
tTau: Total tau
YKL40: Chitinase-3-like protein-1
