# Supplementary material for: Identifying clinically useful biomarkers in neurodegenerative disease through a collaborative approach: the NeuroToolKit
Source: Alzheimers Res Ther. 2023 Jan 28;15:25. doi: 10.1186/s13195-023-01168-y (PMC9883877; doi:10.1186/s13195-023-01168-y)
Supplement: Supplementary file 1 — Additional file 1. Supplementary methods, tables, and figures. [file 13195_2023_1168_MOESM1_ESM.docx]

**SUPPLEMENTARY MATERIALS**

**Identifying clinically useful biomarkers in neurodegenerative disease through a collaborative approach: The NeuroToolKit**

Sterling C. Johnson, Marc Suárez-Calvet, Ivonne Suridjan, Carolina Minguillón, Juan Domingo Gispert, Erin Jonaitis, Agata Michna, Margherita Carboni, Tobias Bittner, Christina Rabe, Gwendlyn Kollmorgen, Henrik Zetterberg and Kaj Blennow

**CONTENTS**

| Supplementary methods | 2 |
| --- | --- |
| **Table S1**. Median, mean, robust SD and SD of Aβ42, Aβ40, and α-Syn in reference groups before and after correction | 7 |
| **Table S2.** Cut-off values determined by two-GMM (amyloid status as defined by Aβ42/Aβ40 ratio) | 8 |
| **Table S3.** Number of samples in ALFA and Wisconsin cohorts for differently defined cut-off values (amyloid status as defined by Aβ42/Aβ40 ratio) – two-GMM | 8 |
| **Table S4.** Cut-off values determined by two-GMM (amyloid status as defined by pTau/Aβ42 ratio) | 8 |
| **Table S5.** Number of samples in ALFA and Wisconsin cohorts for differently defined cut-off values (amyloid status as defined by pTau/Aβ42 ratio) – two-GMM | 8 |
| Supplementary results | 9 |
| **Table S6.** Characterisation of cohorts, including uncorrected and corrected data (pTau/Aβ42 ratio) | 12 |
| **Table S7.** Characterisation of cohorts with corrected data (amyloid status as defined by Aβ42/Aβ40 ratio) using the cut-off value derived from the ALFA cohort | 14 |
| **Table S8.** Fold change for CU individuals | 16 |
| **Table S9.** Fold change for patients with MCI | 18 |
| **Table S10.** Fold change for patients with AD-dementia | 19 |
| **Table S11.** AUC and 95% confidence interval for the ROC curve (amyloid status as defined by Aβ42/Aβ40) | 21 |
| **Figure S1.** Biomarker distributions for uncorrected and corrected (A) Aβ42, (B) Aβ40, and (C) α-Syn in the reference group (i.e. CU individuals who were APOE-ε4 non-carriers and aged <65 years) | 22 |
| **Figure S2.** Baseline correlation between NTK biomarkers in all CU cases ([A] ALFA+ cohort and [B] Wisconsin cohort) and baseline correlation between NTK biomarkers and all AD-dementia cases ([C] Wisconsin cohort and [D] Abby/Blaze cohort) | 24 |
| **Figure S3.** ROC analysis of CU A- individuals compared with CU A+ individuals, patients with MCI A+, and patients with AD-dementia (amyloid status as defined by Aβ42/Aβ40) | 25 |
| References | 26 |

**SUPPLEMENTARY METHODS**

**ALFA cohort:**

Participants [1]

The ALFA (for ALzheimer and FAmilies) parent study (NCT01835717) aimed to characterize preclinical AD in CU individuals, most with a family history of AD and aged 45–75 years. In the ALFA+ nested cohort (NCT02485730), participants underwent a more comprehensive evaluation compared with the parent study, including CSF biomarker analysis. CSF samples (n=398) were obtained by lumbar puncture following a standardized protocol and all measurements were performed at the Clinical Neurochemistry Laboratory, Sahlgrenska University Hospital (Mölndal, Sweden). Eligible participants were cognitively unimpaired (CU) Spanish and/or Catalan-speaking persons aged
45–74 years who agreed with the study procedures and tests, which included clinical interviews and questionnaires associated with risk factors, cognitive tests, a blood sample extraction for DNA analysis, and magnetic resonance imaging. A large proportion of the individuals recruited were CU offspring of patients with Alzheimer’s disease (AD).

Exclusion criteria were:

- Cognitive performance falling outside the established cut-offs (Mini-Mental State Examination [MMSE] <26 [2, 3], or Memory Impairment Screen <6 [4, 5], or Time‑Orientation subtest of the Barcelona Test II <68, or Semantic Fluency [animal naming] <12 [6, 7]).
- Clinical Dementia Rating scale (CDR) >0 [8].
- Major psychiatric disorders (according to DSM-IV-TR), or diseases that could affect cognitive abilities, including mood disorders determined by the Goldberg Anxiety and Depression Scale [9, 10].
- Severe auditory and/or visual disorder, neurodevelopmental, and/or psychomotor disorder.
- Significant diseases that could currently interfere with cognition.
- Neurological disorders, such as Parkinson's disease, stroke, epilepsy under treatment with frequent seizures (>1/month) in the past year, multiple sclerosis, or other serious neurological diseases.
- Brain injury that could interfere with cognition.
- Suspected pattern of family history of autosomal dominant AD: Three affected individuals in two different generations with an onset before the age of 60 years.

Cerebrospinal fluid collection, processing and storage [11]

Cerebrospinal fluid (CSF) samples were obtained by lumbar puncture following standard procedures [12]. Lumbar puncture was performed at the intervertebral space L3/L4, L4/L5 or L5/S1 using a standard needle. All lumbar punctures were performed fasted between 8am and 12pm. CSF was collected into a 15 mL sterile polypropylene tube (Starstedt, Nümbrecht, Germany). CSF was aliquoted into 0.5 mL sterile polypropylene tubes (Starstedt, Nümbrecht, Germany), and frozen at −80°C. Overall, the time between collection and freezing was less than 30 minutes. All the determinations were done in aliquots that had never been previously thawed.

CSF biomarkers measurements [11]

All the measurements were performed at the Clinical Neurochemistry Laboratory, Sahlgrenska University Hospital (Mölndal, Sweden) using the Elecsys® and NeuroToolKit (NTK) immunoassays (Roche Diagnostics International Ltd). Total tau (tTau) and phosphorylated tau (pTau) measurements were performed using the immunoassays Elecsys Total-tau CSF and Phospho-Tau(181P) CSF immunoassays on a fully automated cobas e 601 analyzer (Roche Diagnostics International Ltd). The rest of the biomarkers were measured with the prototype NTK on a cobas e 411 and e 601 analyzers (Roche Diagnostics International Ltd).

**Wisconsin cohort [13]**

Participants

Participating studies included the Wisconsin Registry for Alzheimer’s Prevention study [10], Wisconsin Alzheimer’s Disease Research Center, and the Alzheimer’s Disease Connectome Project. Enrollment criteria varied between studies. These studies included CU individuals, participants with MCI, or AD-dementia, enriched for parental history of AD. CSF samples were obtained by lumbar puncture; all samples were re-assayed following primary analysis at the Clinical Neurochemistry Laboratory, University of Gothenburg (Gothenburg, Sweden). For the purposes of this analysis, the date of the first lumbar puncture was considered the study baseline.

Wisconsin Registry for Alzheimer’s Prevention (WRAP) has been ongoing since late 2001. Participants were recruited from memory clinics in which a parent was diagnosed or treated, media advertisements, community outreach, and word of mouth. Eligible participants were aged 40–65 years at study entry, fluent English speakers, had visual and auditory acuity adequate for neuropsychological testing, and were generally healthy with no diseases expected to interfere with study participation over time. Participants were excluded from enrollment if they had a prior diagnosis of dementia or evidence of dementia at baseline testing. Cognitive testing was completed every two years from Visit 2 onward, (average interval between Visit 1 and Visit 2 was four years).

Wisconsin Alzheimer’s Disease Research Center (ADRC) and Alzheimer’s Disease Connectome Project (ADCP) recruited individuals who were CU, had mild cognitive impairment (MCI), or dementia. ADCP participants were recruited from memory clinics and from the Wisconsin ADRC. Wisconsin ADRC participants were recruited from memory clinics, community lectures, radio and newspaper advertisements, and word of mouth. Eligible participants were aged ≥45 years. Exclusion criteria included major medical, neurologic, or psychiatric conditions that would confound interpretation of cognitive changes. The ADRC cohort completed cognitive testing annually for participants with impairment or CU participants aged >65 years, or every two years for CU participants <65 years. Lumbar punctures were a requirement of enrollment for CU participants and participants with MCI but optional for participants with dementia and participants from underrepresented groups.

CSF collection, processing and storage

CSF samples were obtained by syringe suction (aspirating method). After discarding the first 1 mL and sending 1 mL to the laboratory for cell count, 20 mL of CSF were collected in four 5 mL polypropylene syringes. Samples were combined into a 30 mL polypropylene centrifuge tube (Evergreen Scientific, Los Angeles, CA, USA) then gently mixed prior to being centrifuged for 10 min at 2000 g at 4°C. The sample was then transferred into a second 30 mL polypropylene tube. Following gentle mixing, 0.5 mL CSF fluid was aliquoted into approximately 40 x 1.5 mL polypropylene tubes (Fisher Scientific, Waltham, MA, USA) and frozen at -80°C. Overall, the time between collection and freezing was less than 30 minutes.

CSF biomarkers measurements

All samples were re-assayed following primary analysis at the Clinical Neurochemistry Laboratory, University of Gothenburg. Aβ42, Aβ40, pTau, and tTau were analysed on the same day and exploratory NTK analytes were assayed on separate days. The Elecsys β-amyloid(1-42), Phospho-Tau(181P), and Total-Tau, S100 calcium binding protein B (S100B), and interleukin-6 (IL-6) CSF immunoassays were performed on a cobas e 601 analyzer. The remaining NTK panel immunoassays were measured using a cobas e 411 analyzer.

**Abby/Blaze cohort**

These interventional studies investigated the efficacy and safety of crenezumab compared with placebo for the treatment of mild to moderate AD.

Participants

Patients were eligible to participate in the ABBY [14] and BLAZE [15] studies if they were aged 50–80 years, met the criteria for mild to moderate probable AD according to the National Institute of Neurologic and Communicative Disorders and Stroke/Alzheimer’s Disease and Related Disorders Association criteria [16], and with MMSE score of 18–26 points at the time of screening [2]. Additional inclusion criteria were a Geriatric Depression Scale score of <6, a CDR Sum of Boxes (CDR-SB) score of ≥0.5 [17-19], and an Alzheimer’s Disease Assessment Scale cognitive subscale (ADAS-Cog) Delayed Word Recall score of ≥5 [20]. Patients were required to have evidence of elevated amyloid burden consistent with a diagnosis of AD indicating moderate-to-frequent neuritic plaques (amyloid positive) as assessed by a central expert blinded visual reading of the screening florbetapir PET scan (BLAZE study only). Treatment with approved AD drugs such as acetylcholinesterase inhibitors, or memantine, initiated ≥3 months and stabilized ≥2 months prior to randomisation was permitted.

CSF collection, processing and storage

BLAZE [15]: CSF was collected from all patients at screening and prior to dosing at Week 69, or at early termination/discontinuation if necessary. Samples of 10–12 mL of CSF were collected by lumbar puncture at L4/L5 with a Sprotte atraumatic needle into 15-mL low-retention polypropylene tubes (Sarstedt AG, Numbrecht, Germany), frozen immediately on dry ice, and stored at −80°C. For aliquoting, CSF was thawed on ice, vortexed for 30 s at maximum speed and centrifuged at 2000 g for 3 min. Aliquots (0.5 mL) were dispensed into 0.5-mL low-retention, screw-cap MAXYmum Recovery TM tubes using the corresponding low-retention pipette tips (Axygen Scientific Inc., Union City, CA, US) and frozen at −80°C.

ABBY: The procedure in the ABBY study was the same as the above BLAZE study; however, only a subset of patients in ABBY provided CSF.

CSF biomarkers measurements

All samples were measured at Covance, Indianapolis, USA. Amyloid-β_1–42_ (Aβ42), amyloid-β_1–40_ (Aβ40), pTau, and tTau were analysed on the same day and exploratory NTK analytes were assayed on separate days. The Elecsys β-amyloid(1-42), Phospho-Tau(181P), and Total-Tau, S100 calcium binding protein B (S100B), and interleukin-6 (IL-6) CSF immunoassays were performed on a cobas e 601 analyzer. The remaining NTK panel immunoassays were measured using a cobas e 411 analyzer.

Gaussian mixture modeling analysis

To determine the best model, three-Gaussian mixed modeling (GMM) and four-GMM were also performed, and the Akaike information criterion (AIC) was determined. The best fit (lowest AIC value) for Aβ42/Aβ40 ratio was achieved for the solution with three components for the ALFA cohort and four components for the Wisconsin cohort. However, the cut-off value derived from this model classified only 25% of the individuals from the Wisconsin cohort as A-, a likely low estimate for this patient population. The best fit for pTau/Aβ42 ratio was achieved with three components for the ALFA cohort and two components for the Wisconsin cohort. The GMM with two components was selected for both ratios as there was little gain when using more complicated models (Tables S1 and S3). The number of samples in the ALFA and Wisconsin cohorts with amyloid positivity are shown in Tables S2 and S4. Variations in amyloid status classification were less pronounced in the pTau/Aβ42 ratio analysis as this method is more robust to small changes in cut-off values.

The analysis was performed independently using Gaussian parameters (µ, σ) for both Aβ42/Aβ40 ratio and pTau/Aβ42 and the conclusions did not change. The cut-off values for the pTau/Aβ42 ratio were defined as 0.013 and 0.026 for the ALFA and Wisconsin cohorts, respectively.

**Table S1** Median, mean, robust SD and SD of Aβ42, Aβ40, and α-Syn in reference groups before and after correction

|  |  | **Uncorrected** | | | | **Corrected** | | | |
| --- | --- | --- | --- | --- | --- | --- | --- | --- | --- |
| **Biomarker** | **Cohort** | **Median** | **Mean** | **rSD** | **SD** | **Median** | **Mean** | **rSD** | **SD** |
| Aβ42 | ALFA | 1528 | 1591 | 614.6 | 652.6 | 1528 | 1591 | 614.6 | 652.6 |
|  | Wisconsin | 941.1 | 996.7 | 409.9 | 468.9 | 1528 | 1618 | 659.1 | 761.0 |
| Aβ40 | ALFA | 17.81 | 18.32 | 5.238 | 5.192 | 17.81 | 18.32 | 4.938 | 5.192 |
|  | Wisconsin | 13.64 | 13.90 | 4.471 | 4.641 | 17.81 | 18.16 | 5.838 | 6.060 |
| α-Syn | ALFA | 193.8 | 256.1 | 83.59 | 338.4 | 193.8 | 256.1 | 83.59 | 338.4 |
|  | Wisconsin | 137.8 | 149.9 | 58.57 | 64.04 | 193.8 | 210.8 | 82.38 | 90.06 |

*Abbreviations*: Aβ42, amyloid-β_1–42_; Aβ40, amyloid-β_1–40_; α-Syn, alpha-synuclein; rSD, robust standard deviation; SD, standard deviation

**Table S2** Cut-off values determined by two-GMM (amyloid status as defined by Aβ42/Aβ40 ratio)

| **Cohort** | **µ, σ (mu, sigma)** | **Mean, SD** | **Median, rSD** |
| --- | --- | --- | --- |
| ALFA | 0.071 | 0.073 | 0.071 |
| Wisconsin | 0.060 | 0.064 | 0.063 |

*Abbreviations*: Aβ42, amyloid-β_1–42_; Aβ40, amyloid-β_1–40_; GMM, Gaussian mixed modelling; rSD, robust standard deviation; SD, standard deviation

**Table S3** Number of samples in ALFA and Wisconsin cohorts for differently defined cut-off values (amyloid status as defined by Aβ42/Aβ40 ratio) – two-GMM

| **Cut-off estimator** | **Alfa** | | **Wisconsin** | | | **Wisconsin (c/o ALFA)** | | |
| --- | --- | --- | --- | --- | --- | --- | --- | --- |
|  | **A-** | **A+** | **A-** | **A+** | **NA** | **A-** | **A+** | **NA** |
| mu, sigma | 263 | 135 | 404 | 237 | 10 | 431 | 210 | 10 |
| Mean, SD | 247 | 151 | 353 | 288 | 10 | 416 | 225 | 10 |
| Median, rSD | 263 | 135 | 370 | 271 | 10 | 431 | 210 | 10 |

*Abbreviations*: Aβ42, amyloid-β_1–42_; Aβ40, amyloid-β_1–40_; c/o, cut-off value; GMM, Gaussian mixed modelling; NA, not applicable; rSD, robust standard deviation; SD, standard deviation

**Table S4** Cut-off values determined by two-GMM (amyloid status as defined by pTau/Aβ42 ratio)

| **Cohort** | **µ, σ (mu, sigma)** | **Mean, SD** | **Median, rSD** |
| --- | --- | --- | --- |
| ALFA | 0.013 | 0.013 | 0.013 |
| Wisconsin | 0.026 | 0.026 | 0.027 |

*Abbreviations*: Aβ42, amyloid-β_1–42_; GMM, Gaussian mixed modelling; pTau, phosphorylated tau; rSD, robust standard deviation; SD, standard deviation

**Table S5** Number of samples in ALFA and Wisconsin cohorts for differently defined cut-off values (amyloid status as defined by pTau/Aβ42 ratio) – two-GMM

| **Cut-off estimator** | **Alfa** | | | **Wisconsin** | | | **Wisconsin (c/o ALFA)** | | | |
| --- | --- | --- | --- | --- | --- | --- | --- | --- | --- | --- |
|  | **A-** | **A+** | **NA** | **A-** | **A+** | **NA** | | **A-** | **A+** | **NA** |
| mu, sigma | 248 | 131 | 19 | 458 | 184 | 9 | | 403 | 239 | 9 |
| Mean, SD | 248 | 131 | 19 | 458 | 184 | 9 | | 403 | 239 | 9 |
| Median, rSD | 248 | 131 | 19 | 465 | 177 | 9 | | 403 | 239 | 9 |

*Abbreviations*: Aβ42, amyloid-β_1–42_; c/o, cut-off value; GMM, Gaussian mixed modelling; NA, not applicable; pTau, phosphorylated tau; rSD, robust standard deviation; SD, standard deviation

**SUPPLEMENTARY RESULTS**

## Correlations of CSF biomarker concentrations for CU individuals

Baseline/enrollment values across cohorts in the same disease stage were comparable for all measured biomarkers. The highest correlation values in CU individuals were consistent across the same CSF biomarkers in the two cohorts that included this population (ALFA+ and Wisconsin). Neurogranin strongly correlated with α-Syn (ALFA+: 0.71; Wisconsin: 0.81), Aβ40 (ALFA+: 0.85; Wisconsin: 0.81), pTau (ALFA+: 0.90; Wisconsin: 0.91), and tTau (ALFA+: 0.93; Wisconsin: 0.93). Correlations between α-Syn and Aβ40 (ALFA+: 0.71; Wisconsin: 0.79), pTau (ALFA+: 0.70; Wisconsin: 0.83), and tTau (ALFA+: 0.72; Wisconsin: 0.84) were strong. Aβ40 strongly correlates with Aβ42 (ALFA+: 0.70; Wisconsin: 0.76), pTau (ALFA+: 0.81; Wisconsin: 0.80), and tTau (ALFA+: 0.86; Wisconsin: 0.82). Strong correlation was also found between pTau and tTau (ALFA+: 0.98; Wisconsin: 0.99).

## Correlations of CSF biomarker concentrations for patients with AD-dementia

The highest correlation values in patients with AD-dementia were consistent across the same CSF biomarkers in two cohorts (Wisconsin and Abby/Blaze). Neurogranin strongly correlated with α‑Syn (Wisconsin: 0.76; Abby/Blaze: 0.61), Aβ40 (Wisconsin: 0.78; Abby/Blaze:0.78), pTau (Wisconsin: 0.82; Abby/Blaze: 0.93), and tTau (Wisconsin: 0.82; Abby/Blaze: 0.93). α-Syn strongly correlated with Aβ40 (Wisconsin: 0.83; Abby/Blaze: 0.55), pTau (Wisconsin: 0.76; Abby/Blaze: 0.61), and tTau (Wisconsin: 0.85; Abby/Blaze: 0.64). Aβ40 strongly correlated with Aβ42 (Wisconsin: 0.75; Abby/Blaze: 0.70), pTau (Wisconsin: 0.59; Abby/Blaze: 0.73), tTau (Wisconsin: 0.67; Abby/Blaze: 0.76). pTau and tTau strongly correlated with each other (Wisconsin: 0.93; Abby/Blaze: 0.98). Importantly, the highest correlation values were found across the same biomarkers regardless of disease stage. Figure 3 shows the correlogram matrices of the concentration of all biomarkers in CU individuals and patients with AD-dementia across the cohorts.

**Table S6** Characterisation of cohorts, including uncorrected and corrected data (amyloid status as defined by pTau/Aβ42 ratio)

|  | **CU (A-)** | | | **CU (A+)** | | | **CU (unknown)** | | **MCI (A-)** | **MCI (A+)** | **AD-dementia (all)** | | |
| --- | --- | --- | --- | --- | --- | --- | --- | --- | --- | --- | --- | --- | --- |
| **Characteristics** | **ALFA** | **Wisconsin** | ***p*** | **ALFA** | **Wisconsin** | ***p*** | **ALFA** | **Wisconsin** | **Wisconsin** | **Wisconsin** | **Wisconsin** | Abby/Blaze | **p** |
| *N* | 248 | 431 |  | 131 | 106 |  | 19 | 9 | 23 | 33 | 49 | 164 |  |
| Age, mean (SD) | 60.3 (4.5) | 60.5 (7.4) |  | 62.7 (4.9) | 65.1 (7.3) |  | 61.1 (4.7) | 62.1 (6.6) | 68.8 (8.2) | 73.9 (7.9) | 72.3 (8.5) | 69.6 (7.8) |  |
| Female, *n* (%) | 159 (40) | 286 (43) |  | 78 (19) | 72 (11) |  | 7 (2) | 7 (1) | 11 (2) | 13 (2) | 17 (3) | 85 (52) |  |
| Education, mean (SD) | 13.5 (3.5) | 16.2 (2.5) |  | 13.4 (3.7) | 16.5 (2.4) |  | 14.5 (3.5) | 16.8 (3.2) | 15.5 (2.8) | 16.3 (2.5) | 14.5 (2.7) | NA |  |
| MMSE, mean (SD) | 29.2 (0.9) | 29.4 (0.8) |  | 29.1 (1.0) | 29.2 (1.1) |  | 29.2 (0.9) | 29.3 (1.0) | 28.2 (1.8) | 27.0 (2.2) | 21.7 (3.8) | 21.7 (3.2) |  |
| CDR-SB, mean (SD) | 0 | 0.05 (0.20) |  | 0 | 0.14 (0.34) |  | 0 | 0 | 1.35 (1.40) | 1.83 (1.20) | 4.44 (1.61) | 4.56 (1.94) |  |
| PACC, mean (SD) | -0.14 (0.94) | -0.14 (1.15) |  | -0.16 (1.04) | -0.40 (1.28) |  | -0.06 (0.80) | -0.21 (0.97) | -2.22 (1.02) | -2.95 (0.96) | -4.60 (1.42) | NA |  |
| APOE4 carriers, *n* (%) | 106 (27) | 134 (21) |  | 97 (24) | 57 (9) |  | 11 (3) | 4 (1) | 6 (1) | 20 (3) | 32 (5) | 118 (72) |  |
| APOE4 non-carriers, *n* (%) | 142 (36) | 274 (42) |  | 34 (9) | 45 (7) |  | 8 (2) | 5 (1) | 13 (2) | 12 (2) | 16 (3) | 46 (28) |  |
| Uncorrected CSF biomarkers, median (IQR) | | | | | | | | | | | | | |
| YKL40, ng/mL | 137.1 (58.4) | 129.8 (58.3) | 0.359 | 154.7 (73.0) | 146.6 (67.5) | 0.747 | 100.6 (21.3) | 161.9 (44.1) | 152.1 (91.8) | 223.2 (96.8) | 216.4 (129.2) | 191.6 (103.9) | 0.112 |
| sTREM2, ng/mL | 7.60 (2.70) | 7.49 (3.15) | 0.461 | 7.99 (2.79) | 8.37 (3.34) | 0.922 | 5.65 (1.58) | 9.25 (2.63) | 8.11 (2.51) | 9.81 (4.39) | 9.36 (3.74) | 8.93 (4.15) | 0.844 |
| GFAP, ng/mL | 7.02 (2.92) | 8.32 (4.24) | <0.001 | 8.14 (3.64) | 9.29 (4.30) | <0.001 | 6.02 (2.13) | 9.18 (3.71) | 9.02 (5.54) | 14.33 (7.19) | 13.62 (6.24) | 10.58 (6.27) | 0.002 |
| IL-6, pg/mL | 3.58 (1.47) | 3.96 (2.02) | <0.001 | 3.39 (1.87) | 3.75 (1.67) | 0.397 | 4.46 (2.33) | NA | 3.52 (1.38) | 3.33 (1.21) | 4.17 (1.78) | 3.08 (1.52) | <0.001 |
| NfL, pg/mL | 75.4 (31.6) | 77.3 (39.6) | 0.139 | 86.7 (35.0) | 94.3 (56.8) | 0.080 | 69.4 (22.4) | 66.9 (35.5) | 106.6(113.9) | 160.1(100.7) | 195.2(137.4) | 189.6 (82.6) | 0.908 |
| Neurogranin, pg/mL | 695.8 (336.8) | 692.2 (364.4) | 0.327 | 852.5 (501.3) | 890.1 (545.3) | 0.268 | 351.4 (51.1) | 820.2 (377.6) | 660.0 (314.1) | 928.1 (620.2) | 1006.0 (362.9) | 999.9  (757.9) | 0.908 |
| S100, ng/mL | 0.97 (0.29) | 1.12 (0.31) | <0.001 | 1.08 (0.35) | 1.12 (0.37) | 0.071 | 0.91 (0.23) | 1.15 (0.00) | 1.01 (0.33) | 1.31 (0.48) | 1.18 (0.33) | 1.10 (0.32) | 0.038 |
| α-Syn, pg/mL | 181.0 (96.7) | 140.3 (72.8) | <0.001 | 202.4 (107.4) | 175.4 (109.1) | 0.010 | 96.8  (48.1) | 176.5 (68.3) | 142.7 (115.1) | 226.1 (118.7) | 229.2  (134.3) | 379.5  (245.6) | <0.001 |
| Aβ40, ng/mL | 17.1 (6.3) | 14.0 (6.1) | <0.001 | 17.0 (6.6) | 13.7 (5.8) | <0.001 | 9.0 (3.6) | NA | 14.1 (6.6) | 15.1 (7.4) | 13.1 (6.4) | 15.8 (6.7) | 0.007 |
| Aβ42, pg/mL | 1434.5 (741.8) | 997.1 (522.9) | <0.001 | 818.8 (353.7) | 501.9 (247.2) | <0.001 | 698.4 (302.0) | 662.2 (0.0) | 934.9 (689.7) | 459.9 (214.3) | 401.9  (243.3) | 576.8  (234.5) | <0.001 |
| pTau, pg/mL | 13.8 (6.6) | 15.1 (6.9) | <0.001 | 17.9 (10.1) | 21.8 (10.6) | 0.006 | NA | NA | 14.6 (6.3) | 27.9 (15.4) | 35.6 (18.9) | 35.5 (23.0) | 0.908 |
| tTau, pg/mL | 173.8 (65.8) | 173.2 (75.8) | 0.950 | 216.9 (107.8) | 236.6 (113.7) | 0.085 | 104.7 (11.4) | 136.2  (0.0) | 173.7 (104.8) | 304.9 (171.5) | 351.7  (135.9) | 345.6  (201.9) | 0.908 |
| pTau/Aβ42 | 0.0093 (0.0021) | 0.0156 (0.0056) | <0.001 | 0.0205 (0.0121) | 0.0399 (0.0196) | <0.001 | NA | NA | 0.0153 (0.0038) | 0.0612 (0.0466) | 0.0860 (0.0565) | 0.0590 (0.0293) | <0.001 |
| Aβ42/Aβ40 | 0.0862 (0.0144) | 0.0720 (0.0145) | <0.001 | 0.0518 (0.0201) | 0.0391 (0.0132) | <0.001 | 0.0811 (0.0060) | NA | 0.0725 (0.0079) | 0.0303 (0.0083) | 0.0308 (0.0120) | 0.0389 (0.0121) | <0.001 |
| Corrected CSF biomarkers, median (IQR) | | | | | | | | | | | | | |
| α-Syn, pg/mL | 181.0 (96.7) | 197.3 (102.4) | 0.073 | 202.4 (107.4) | 246.7 (153.4) | 0.007 | 96.8 (48.1) | 248.2 (96.1) | 200.7 (161.9) | 318.0 (166.9) | 322.3  (188.9) | 379.5 (245.6) | 0.109 |
| Aβ40, ng/mL | 17.1 (6.3) | 18.3 (8.0) | 0.073 | 17.0 (6.6) | 17.8 (7.6) | 0.399 | 9.0 (3.6) | NA | 18.5 (8.6) | 19.7 (9.7) | 17.1 (8.3) | 15.8 (6.7) | 0.250 |
| Aβ42, pg/mL | 1434.5 (741.8) | 1618.4 (848.6) | 0.021 | 818.8 (353.7) | 814.6 (401.2) | 0.713 | 698.4 (302.0) | 1074.8 (0.0) | 1517.4 (1119.5) | 746.5 (347.8) | 652.3 (394.9) | 576.8 (234.5) | 0.250 |
| pTau/Aβ42 | 0.0093 (0.0021) | 0.0096 (0.0035) | 0.359 | 0.0205 (0.0121) | 0.0246 (0.0121) | 0.002 | NA | NA | 0.0094 (0.0023) | 0.0377 (0.0287) | 0.0530 (0.0348) | 0.0590 (0.0293) | 0.256 |
| Aβ42/Aβ40 | 0.0862 (0.0144) | 0.0895 (0.0180) | 0.014 | 0.0518 (0.0201) | 0.0486 (0.0164) | 0.290 | 0.0811 (0.0060) | NA | 0.0901 (0.0099) | 0.0377 (0.0103) | 0.0383 (0.0149) | 0.0389 (0.0121) | 0.908 |

*p*-values are adjusted using FDR method for each cohort, amyloid-β status, and disease stage comparison independently.

*Abbreviations*: Aβ42, amyloid-β_1–42_; Aβ40, amyloid-β_1–40_; α-Syn, alpha-synuclein; AD, Alzheimer’s disease; CDR-SB, Clinical Dementia Rating Scale – Sum of Boxes; CU, cognitively unimpaired; CSF, cerebrospinal fluid; FDR, False Discovery Rate; GFAP, glial fibrillary acidic protein; IL, interleukin; IQR, interquartile range; MCI, mild cognitive impairment; MMSE, Mini Mental State Examination; NfL, neurofilament light; PACC, Preclinical Alzheimer’s Cognitive Composite; pTau, phosphorylated tau; rSD, robust standard deviation; SD, standard deviation; sTREM2, soluble triggering receptor expressed on myeloid cells 2; tTau, total tau; YKL40, chitinase-3-like protein-1

**Table S7** Characterisation of cohorts with corrected data (amyloid status as defined by Aβ42/Aβ40 ratio) using the cut-off value derived from the ALFA cohort

|  | **CU (A-)** | | | | **CU (A+)** | | | **CU (unknown)** | **MCI (A-)** | **MCI (A+)** | **AD-dementia (all)** | | |
| --- | --- | --- | --- | --- | --- | --- | --- | --- | --- | --- | --- | --- | --- |
| **Characteristics** | **ALFA** | **Wisconsin** | ***p*** | **ALFA** | | **Wisconsin** | ***p*** | **Wisconsin** | **Wisconsin** | **Wisconsin** | **Wisconsin** | **Abby/Blaze** | **p** |
| *N* | 263 | 403 |  | 135 | | 133 |  | 10 | 23 | 33 | 49 | 164 |  |
| Age, mean (SD) | 60.5 (4.5) | 60.4 (7.5) |  | 62.2 (5.0) | | 64.7 (6.9) |  | 60.7 (7.6) | 69.2 (8.5) | 73.7 (7.9) | 72.3 (8.5) | 69.6 (7.8) |  |
| Female, *n* (%) | 163 (41) | 267 (41) |  | 81 (20) | | 90 (14) |  | 8 (1) | 12 (2) | 12 (2) | 17 (3) | 85 (52) |  |
| Education, mean (SD) | 13.6 (3.5) | 16.2 (2.5) |  | 13.3 (3.6) | | 16.3 (2.4) |  | 17.1 (3.1) | 15.7 (2.7) | 16.2 (2.6) | 14.5 (2.7) | NA |  |
| MMSE, mean (SD) | 29.2 (0.9) | 29.4 (0.8) |  | 29.1 (1.0) | | 29.2 (1.1) |  | 29.3 (1.0) | 28.2 (1.7) | 27.0 (2.2) | 21.7 (3.8) | 21.7 (3.2) |  |
| CDR-SB, mean (SD) | 0 | 0.05 (0.20) |  | 0 | | 0.12 (0.31) |  | 0 | 1.38 (1.44) | 1.81 (1.18) | 4.44 (1.61) | 4.56 (1.94) |  |
| PACC, mean (SD) | -0.16 (0.93) | -0.14 (1.15) |  | -0.11 (1.03) | | -0.35 (1.28) |  | -0.02 (0.98) | -2.26 (1.04) | -2.93 (0.97) | -4.60 (1.42) | NA |  |
| APOE4 carriers, *n* (%) | 111 (28) | 116 (18) |  | 103 (26) | | 75 (12) |  | 4 (1) | 6 (1) | 20 (3) | 32 (5) | 118 (72) |  |
| APOE4 non-carriers, *n* (%) | 152 (38) | 267 (41) |  | 32 (8) | | 51 (8) |  | 6 (1) | 13 (2) | 12 (2) | 16 (3) | 46 (28) |  |
| CSF biomarkers, median (IQR) | | | | | | | | | | | | | |
| YKL40, ng/mL | 136.2 (60.6) | 130.0 (58.6) | 0.122 | 147.6 (70.6) | | 143.1 (69.0) | 0.973 | 154.8 (64.1) | 152.1 (104.5) | 222.0 (96.8) | 216.4 (129.2) | 191.6 (103.9) | 0.201 |
| sTREM2, ng/mL | 7.57 (2.74) | 7.45 (3.24) | 0.092 | 7.58 (3.05) | | 8.43 (3.36) | 0.366 | 9.07 (2.59) | 8.11 (2.24) | 9.81 (4.39) | 9.36 (3.74) | 8.93 (4.15) | 0.908 |
| GFAP, ng/mL | 7.00 (2.93) | 8.14 (4.19) | <0.001 | 8.02 (3.43) | | 9.33 (4.02) | <0.001 | 8.72 (3.75) | 9.02 (5.54) | 14.33 (5.72) | 13.62 (6.24) | 10.58 (6.27) | 0.006 |
| IL-6, pg/mL | 3.57 (1.58) | 3.98 (2.03) | <0.001 | 3.52 (1.71) | | 3.75 (1.77) | 0.582 | NA | 3.52 (1.38) | 3.33 (1.21) | 4.17 (1.78) | 3.08 (1.52) | <0.001 |
| NfL, pg/mL | 75.6 (31.9) | 75.5 (39.9) | 0.567 | 86.2 (36.0) | | 93.2 (44.6) | 0.038 | 66.6 (34.7) | 106.6 (116.2) | 151.0 (98.8) | 195.2 (137.4) | 189.6 (82.6) | 0.908 |
| Neurogranin, pg/mL | 683.7 (360.7) | 681.7 (357.9) | 0.077 | 805.9 (466.4) | | 891.8 (518.1) | 0.160 | 805.7 (439.3) | 666.8 (285.4) | 928.1 (620.2) | 1006.0 (362.9) | 999.9 (757.9) | 0.908 |
| S100, ng/mL | 0.97 (0.29) | 1.12 (0.32) | <0.001 | 1.06 (0.38) | | 1.15 (0.33) | 0.002 | 0.92 (0.23) | 1.09 (0.35) | 1.30 (0.47) | 1.18 (0.33) | 1.10 (0.32) | 0.101 |
| α-Syn, pg/mL | 179.4 (99.6) | 195.4 (103.1) | 0.383 | 190.5 (101.7) | | 242.5 (150.9) | <0.001 | 238.5 (100.2) | 200.7 (161.9) | 318.0 (170.2) | 322.3 (188.9) | 379.5 (245.6) | 0.109 |
| Aβ40, ng/mL | 16.6 (7.0) | 18.0 (7.5) | 0.212 | 16.9 (6.0) | | 18.7 (8.6) | 0.038 | NA | 18.5 (9.1) | 19.7 (9.0) | 17.1 (8.3) | 15.8 (6.7) | 0.250 |
| Aβ42, pg/mL | 1417.0 (779.5) | 1631.2 (877.8) | 0.032 | 821.6 (366.0) | | 919.5 (486.6) | 0.061 | 699.7 (375.1) | 1517.4 (1119.5) | 746.5 (347.8) | 652.3 (394.9) | 576.8 (234.5) | 0.250 |
| pTau, pg/mL | 13.8 (6.6) | 14.9 (6.8) | 0.063 | 16.8 (10.0) | | 21.3 (9.9) | 0.002 | 8.0 (0.0) | 14.6 (6.3) | 27.9 (15.4) | 35.6 (18.9) | 35.5 (23.0) | 0.908 |
| tTau, pg/mL | 171.0 (71.1) | 171.6 (73.9) | 0.360 | 209.5 (105.4) | | 235.1 (108.1) | 0.047 | 108.1 (28.1) | 175.1 (108.4) | 304.9 (171.5) | 351.7 (135.9) | 345.6 (201.9) | 0.908 |
| pTau/Aβ42 | 0.0093 (0.0022) | 0.0094 (0.0032) | 0.621 | 0.0199 (0.0127) | | 0.0208 (0.0127) | 0.467 | 0.0246 (0.0000) | 0.0094 (0.0023) | 0.0377 (0.0287) | 0.0530 (0.0348) | 0.0590 (0.0293) | 0.256 |
| Aβ42/Aβ40 | 0.0857 (0.0123) | 0.0904 (0.0157) | <0.001 | 0.0523 (0.0188) | | 0.0533 (0.0203) | 0.467 | NA | 0.0907 (0.0091) | 0.0377 (0.0103) | 0.0383 (0.0149) | 0.0389 (0.0121) | 0.908 |

p-values are adjusted using FDR method for each cohort, amyloid-β status, and disease stage comparison independently.

*Abbreviations*: Aβ42, amyloid-β_1–42_; Aβ40, amyloid-β_1–40_; α-Syn, alpha-synuclein; AD, Alzheimer’s disease; CDR-SB, Clinical Dementia Rating Scale – Sum of Boxes; CU, cognitively unimpaired; CSF, cerebrospinal fluid; FDR, False Discovery Rate; GFAP, glial fibrillary acidic protein; IL, interleukin; IQR, interquartile range; MCI, mild cognitive impairment; MMSE, Mini Mental State Examination; NfL, neurofilament light; PACC, Preclinical Alzheimer’s Cognitive Composite; pTau, phosphorylated tau; rSD, robust standard deviation; SD, standard deviation; sTREM2, soluble triggering receptor expressed on myeloid cells 2; tTau, total tau; YKL40, chitinase-3-like protein-1

**Table S8** Fold change for CU individuals

| **Biomarker** | **Case** | | | | | **Control** | | | | | **Fold change** | | ***p*-value** |
| --- | --- | --- | --- | --- | --- | --- | --- | --- | --- | --- | --- | --- | --- |
|  | **N** | **Median** | **Mean** | **rSD** | **SD** | **N** | **Median** | **Mean** | **rSD** | **SD** | **FC** | **CI** |  |
| ALFA+ A+, control: CU A- Alfa | | | | | | | | | | | | | |
| YKL40 | 135 | 147.6 | 156.6 | 57.3 | 59.4 | 218 | 128.7 | 136.3 | 43.3 | 43.3 | 1.15 | [1.06–1.24] | 0.007 |
| sTREM2 | 135 | 7.58 | 8.03 | 2.63 | 2.40 | 218 | 7.49 | 7.74 | 2.02 | 2.13 | 1.01 | [0.95–1.08] | 0.781 |
| GFAP | 135 | 8.02 | 8.13 | 2.47 | 2.50 | 218 | 6.77 | 7.28 | 2.17 | 2.41 | 1.18 | [1.11–1.27] | 0.003 |
| IL-6 | 135 | 3.52 | 4.23 | 1.42 | 2.37 | 218 | 3.63 | 4.00 | 1.09 | 1.83 | 0.97 | [0.89–1.05] | 0.861 |
| NfL | 135 | 86.2 | 91.3 | 28.4 | 32.5 | 218 | 74.1 | 75.3 | 22.6 | 23.2 | 1.16 | [1.09–1.25] | <0.001 |
| Neurogranin | 135 | 805.9 | 893.5 | 351.3 | 374.9 | 218 | 705.8 | 761.5 | 256.3 | 294.7 | 1.14 | [1.05–1.25] | 0.019 |
| S100 | 135 | 1.06 | 1.07 | 0.25 | 0.27 | 218 | 0.96 | 0.99 | 0.21 | 0.21 | 1.11 | [1.05–1.16] | 0.031 |
| α-Syn | 135 | 190.5 | 251.8 | 75.0 | 329.7 | 218 | 187.7 | 234.8 | 81.0 | 222.4 | 1.02 | [0.93–1.11] | 0.770 |
| Aβ40 | 135 | 16.9 | 17.5 | 4.61 | 4.69 | 218 | 16.8 | 17.5 | 5.27 | 5.10 | 1.00 | [0.94–1.07] | 0.345 |
| Aβ42 | 135 | 821.6 | 893.2 | 274.8 | 309.8 | 218 | 1451.0 | 1564.1 | 560.6 | 591.3 | 0.57 | [0.52–0.61] | <0.001 |
| pTau | 134 | 16.8 | 19.7 | 7.63 | 10.0 | 204 | 14.0 | 15.0 | 4.74 | 4.94 | 1.20 | [1.10–1.31] | <0.001 |
| tTau | 135 | 209.5 | 228.2 | 80.3 | 89.4 | 217 | 172.7 | 182.7 | 54.3 | 56.3 | 1.21 | [1.12–1.31] | <0.001 |
| ALFA+ A+, control: CU A- Wisconsin | | | | | | | | | | | | | |
| YKL40 | 135 | 147.6 | 156.6 | 57.3 | 59.4 | 284 | 125.2 | 127.6 | 36.6 | 39.0 | 1.18 | [1.10–1.27] | <0.001 |
| sTREM2 | 135 | 7.58 | 8.03 | 2.63 | 2.40 | 284 | 7.28 | 7.48 | 2.13 | 2.13 | 1.04 | [0.97–1.11] | 0.052 |
| GFAP | 135 | 8.02 | 8.13 | 2.47 | 2.50 | 284 | 7.78 | 8.20 | 2.66 | 2.69 | 1.03 | [0.97–1.10] | 0.891 |
| IL-6 | 135 | 3.52 | 4.23 | 1.42 | 2.37 | 284 | 4.00 | 4.88 | 1.54 | 3.76 | 0.88 | [0.81–0.95] | 0.012 |
| NfL | 135 | 86.2 | 91.3 | 28.4 | 32.5 | 284 | 69.6 | 78.5 | 23.9 | 61.6 | 1.24 | [1.16–1.33] | <0.001 |
| Neurogranin | 135 | 805.9 | 893.5 | 351.3 | 374.9 | 284 | 682.4 | 728.3 | 253.5 | 283.1 | 1.18 | [1.08–1.29] | <0.001 |
| S100 | 135 | 1.06 | 1.07 | 0.25 | 0.27 | 284 | 1.12 | 1.15 | 0.26 | 0.32 | 0.95 | [0.90–0.99] | 0.006 |
| α-Syn | 135 | 190.5 | 251.8 | 75.0 | 329.7 | 284 | 193.1 | 208.5 | 81.2 | 85.0 | 0.99 | [0.91–1.07] | 0.640 |
| Aβ40 | 135 | 16.9 | 17.5 | 4.61 | 4.69 | 284 | 17.9 | 18.4 | 5.57 | 6.03 | 0.94 | [0.89–1.00] | 0.291 |
| Aβ42 | 135 | 821.6 | 893.2 | 274.8 | 309.8 | 284 | 1620.8 | 1753.0 | 588.6 | 768.6 | 0.51 | [0.47–0.54] | <0.001 |
| pTau | 134 | 16.8 | 19.7 | 7.63 | 10.0 | 284 | 14.6 | 15.5 | 4.96 | 4.99 | 1.15 | [1.06–1.25] | <0.001 |
| tTau | 135 | 209.5 | 228.2 | 80.3 | 89.4 | 284 | 168.1 | 179.7 | 54.9 | 60.8 | 1.25 | [1.16–1.34] | <0.001 |
| Wisconsin A+, control: CU A- Wisconsin | | | | | | | | | | | | | |
| YKL40 | 160 | 138.0 | 155.9 | 59.4 | 63.6 | 284 | 125.2 | 127.6 | 36.6 | 39.0 | 1.10 | [1.02–1.19] | <0.001 |
| sTREM2 | 160 | 7.96 | 8.14 | 2.30 | 2.56 | 284 | 7.28 | 7.48 | 2.13 | 2.13 | 1.09 | [1.03–1.16] | 0.018 |
| GFAP | 160 | 8.84 | 9.55 | 3.36 | 3.56 | 284 | 7.78 | 8.20 | 2.66 | 2.69 | 1.14 | [1.06–1.22] | <0.001 |
| IL-6 | 160 | 3.83 | 4.38 | 1.46 | 2.78 | 284 | 4.00 | 4.88 | 1.54 | 3.76 | 0.96 | [0.89–1.03] | 0.115 |
| NfL | 160 | 90.8 | 101.9 | 38.0 | 49.7 | 284 | 69.6 | 78.5 | 23.9 | 61.6 | 1.31 | [1.21–1.41] | <0.001 |
| Neurogranin | 160 | 843.7 | 898.8 | 385.7 | 374.3 | 284 | 682.4 | 728.3 | 253.5 | 283.1 | 1.24 | [1.14–1.34] | <0.001 |
| S100 | 160 | 1.12 | 1.15 | 0.24 | 0.26 | 284 | 1.12 | 1.15 | 0.26 | 0.32 | 1.00 | [0.96–1.04] | 0.787 |
| α-Syn | 160 | 228.7 | 247.7 | 109.2 | 108.9 | 284 | 193.1 | 208.5 | 81.2 | 85.0 | 1.18 | [1.08–1.29] | <0.001 |
| Aβ40 | 160 | 17.8 | 18.8 | 6.07 | 6.53 | 284 | 17.9 | 18.4 | 5.57 | 6.03 | 0.99 | [0.93–1.06] | 0.605 |
| Aβ42 | 160 | 962.3 | 1034.3 | 414.4 | 431.7 | 284 | 1620.8 | 1753.0 | 588.6 | 768.6 | 0.59 | [0.55–0.64] | <0.001 |
| pTau | 160 | 20.0 | 20.9 | 8.22 | 8.87 | 284 | 14.6 | 15.5 | 4.96 | 4.99 | 1.36 | [1.26–1.47] | <0.001 |
| tTau | 160 | 223.1 | 231.4 | 83.5 | 86.7 | 284 | 168.1 | 179.7 | 54.9 | 60.8 | 1.33 | [1.24–1.42] | <0.001 |
| Wisconsin A+, control: CU A- ALFA+ | | | | | | | | | | | | | |
| YKL40 | 160 | 138.0 | 155.9 | 59.4 | 63.6 | 218 | 128.7 | 136.3 | 43.3 | 43.3 | 1.07 | [0.99–1.16] | 0.065 |
| sTREM2 | 160 | 7.96 | 8.14 | 2.30 | 2.56 | 218 | 7.49 | 7.74 | 2.02 | 2.13 | 1.06 | [1.00–1.13] | 0.541 |
| GFAP | 160 | 8.84 | 9.55 | 3.36 | 3.56 | 218 | 6.77 | 7.28 | 2.17 | 2.41 | 1.31 | [1.21–1.40] | <0.001 |
| IL-6 | 160 | 3.83 | 4.38 | 1.46 | 2.78 | 218 | 3.63 | 4.00 | 1.09 | 1.83 | 1.05 | [0.98–1.13] | 0.171 |
| NfL | 160 | 90.8 | 101.9 | 38.0 | 49.7 | 218 | 74.1 | 75.3 | 22.6 | 23.2 | 1.23 | [1.14–1.32] | <0.001 |
| Neurogranin | 160 | 843.7 | 898.8 | 385.7 | 374.3 | 218 | 705.8 | 761.5 | 256.3 | 294.7 | 1.20 | [1.10–1.30] | 0.014 |
| S100 | 160 | 1.12 | 1.15 | 0.24 | 0.26 | 218 | 0.96 | 0.99 | 0.21 | 0.21 | 1.17 | [1.12–1.22] | <0.001 |
| α-Syn | 160 | 228.7 | 247.7 | 109.2 | 108.9 | 218 | 187.7 | 234.8 | 81.0 | 222.4 | 1.22 | [1.11–1.34] | 0.002 |
| Aβ40 | 160 | 17.8 | 18.8 | 6.07 | 6.53 | 218 | 16.8 | 17.5 | 5.27 | 5.10 | 1.06 | [0.99–1.13] | 0.527 |
| Aβ42 | 160 | 962.3 | 1034.3 | 414.4 | 431.7 | 218 | 1451.0 | 1564.1 | 560.7 | 591.3 | 0.66 | [0.61–0.72] | <0.001 |
| pTau | 160 | 20.0 | 20.9 | 8.22 | 8.87 | 204 | 14.0 | 15.0 | 4.74 | 4.94 | 1.42 | [1.31–1.54] | <0.001 |
| tTau | 160 | 223.1 | 231.4 | 83.5 | 86.7 | 217 | 172.7 | 182.7 | 54.3 | 56.3 | 1.29 | [1.20–1.39] | <0.001 |

*p*-values are adjusted using FDR method for each cohort, amyloid-β status, and disease stage comparison independently.

*Abbreviations*: Aβ42, amyloid-β_1–42_; Aβ40, amyloid-β_1–40_; α-Syn, alpha-synuclein; CU, cognitively unimpaired; FDR, False Discovery Rate; GFAP, glial fibrillary acidic protein; IL, interleukin; NfL, neurofilament light; pTau, phosphorylated tau; rSD, robust standard deviation; SD, standard deviation; sTREM2, soluble triggering receptor expressed on myeloid cells 2; tTau, total tau; YKL40, chitinase-3-like protein-1

**Table S9** Fold change for patients with MCI

| **Biomarker** | **Case** | | | | | **Control** | | | | | **Fold change** | | ***p*-value** |
| --- | --- | --- | --- | --- | --- | --- | --- | --- | --- | --- | --- | --- | --- |
|  | **N** | **Median** | **Mean** | **rSD** | **SD** | **N** | **Median** | **Mean** | **rSD** | **SD** | **FC** | **CI** |  |
| Wisconsin A+, control: CU A- Wisconsin | | | | | | | | | | | | | |
| YKL40 | 33 | 222.0 | 226.4 | 79.3 | 80.9 | 284 | 125.2 | 127.6 | 36.6 | 39.0 | 1.77 | [1.56–2.01] | <0.001 |
| sTREM2 | 33 | 9.81 | 10.3 | 3.41 | 3.48 | 284 | 7.28 | 7.48 | 2.13 | 2.13 | 1.35 | [1.19–1.52] | <0.001 |
| GFAP | 33 | 14.3 | 15.4 | 4.97 | 4.61 | 284 | 7.78 | 8.20 | 2.66 | 2.69 | 1.84 | [1.63–2.09] | <0.001 |
| IL-6 | 33 | 3.33 | 3.78 | 1.28 | 1.60 | 284 | 4.00 | 4.88 | 1.54 | 3.76 | 0.83 | [0.72–0.96] | 0.017 |
| NfL | 33 | 151.0 | 196.0 | 72.5 | 130.3 | 284 | 69.6 | 78.5 | 23.9 | 61.6 | 2.17 | [1.83–2.57] | <0.001 |
| Neurogranin | 33 | 928.1 | 1074.1 | 400.2 | 486.6 | 284 | 682.4 | 728.3 | 253.5 | 283.1 | 1.36 | [1.17–1.59] | <0.001 |
| S100 | 33 | 1.30 | 1.32 | 0.32 | 0.32 | 284 | 1.12 | 1.15 | 0.26 | 0.32 | 1.16 | [1.06–1.27] | 0.002 |
| α-Syn | 33 | 318.0 | 327.1 | 121.0 | 129.1 | 284 | 193.1 | 208.5 | 81.2 | 85.0 | 1.65 | [1.43–1.89] | <0.001 |
| Aβ40 | 33 | 19.69 | 20.6 | 7.01 | 7.17 | 284 | 17.9 | 18.4 | 5.57 | 6.03 | 1.10 | [0.97–1.25] | 0.102 |
| Aβ42 | 33 | 746.5 | 814.9 | 249.8 | 361.7 | 284 | 1620.8 | 1753.0 | 588.6 | 768.6 | 0.46 | [0.41–0.52] | <0.001 |
| pTau | 33 | 27.9 | 34.3 | 15.2 | 17.7 | 284 | 14.6 | 15.5 | 4.96 | 4.99 | 1.91 | [1.58–2.30] | <0.001 |
| tTau | 33 | 304.9 | 346.9 | 132.9 | 147.3 | 284 | 168.1 | 179.7 | 54.9 | 60.8 | 1.81 | [1.56–2.12] | <0.001 |
| Wisconsin A+, control: CU A- ALFA+ | | | | | | | | | | | | | |
| YKL40 | 33 | 222.00 | 226.4 | 79.3 | 80.9 | 218 | 128.7 | 136.3 | 43.3 | 43.3 | 1.72 | [1.51–1.96] | <0.001 |
| sTREM2 | 33 | 9.81 | 10.3 | 3.41 | 3.48 | 218 | 7.49 | 7.74 | 2.02 | 2.13 | 1.31 | [1.16–1.48] | <0.001 |
| GFAP | 33 | 14.3 | 15.4 | 4.97 | 4.61 | 218 | 6.77 | 7.28 | 2.17 | 2.41 | 2.12 | [1.87–2.40] | <0.001 |
| IL-6 | 33 | 3.33 | 3.78 | 1.28 | 1.60 | 218 | 3.63 | 4.00 | 1.09 | 1.83 | 0.92 | [0.80–1.05] | 0.320 |
| NfL | 33 | 151.0 | 196.0 | 72.5 | 130.3 | 218 | 74.1 | 75.3 | 22.6 | 23.2 | 2.04 | [1.72–2.41] | <0.001 |
| Neurogranin | 33 | 928.1 | 1074.1 | 400.2 | 486.6 | 218 | 705.8 | 761.5 | 256.3 | 294.7 | 1.32 | [1.13–1.54] | <0.001 |
| S100 | 33 | 1.30 | 1.32 | 0.32 | 0.32 | 218 | 0.96 | 0.99 | 0.21 | 0.21 | 1.36 | [1.24–1.48] | <0.001 |
| α-Syn | 33 | 318.0 | 327.1 | 121.0 | 129.1 | 218 | 187.7 | 234.8 | 80.9 | 222.4 | 1.69 | [1.47–1.95] | <0.001 |
| Aβ40 | 33 | 19.7 | 20.6 | 7.01 | 7.17 | 218 | 16.8 | 17.5 | 5.27 | 5.10 | 1.17 | [1.03–1.33] | 0.098 |
| Aβ42 | 33 | 746.5 | 814.9 | 249.8 | 361.7 | 218 | 1451.0 | 1564.1 | 560.6 | 591.3 | 0.51 | [0.45–0.58] | <0.001 |
| pTau | 33 | 27.9 | 34.3 | 15.2 | 17.7 | 204 | 14.0 | 15.03 | 4.74 | 4.94 | 1.99 | [1.65–2.41] | <0.001 |
| tTau | 33 | 304.9 | 346.9 | 132.9 | 147.3 | 217 | 172.7 | 182.7 | 54.3 | 56.3 | 1.77 | [1.51–2.06] | <0.001 |

*p*-values are adjusted using FDR method for each cohort, amyloid-β status, and disease stage comparison independently.

*Abbreviations*: Aβ42, amyloid-β_1–42_; Aβ40, amyloid-β_1–40_; α-Syn, alpha-synuclein; CU, cognitively unimpaired; FDR, False Discovery Rate; GFAP, glial fibrillary acidic protein; IL, interleukin; MCI, mild cognitive impairment; NfL, neurofilament light; pTau, phosphorylated tau; rSD, robust standard deviation; SD, standard deviation; sTREM2, soluble triggering receptor expressed on myeloid cells 2; tTau, total tau; YKL40, chitinase-3-like protein-1

**Table S10** Fold change for patients with AD-dementia

| **Biomarker** | **Case** | | | | | **Control** | | | | | **Fold change** | | ***p*-value** |
| --- | --- | --- | --- | --- | --- | --- | --- | --- | --- | --- | --- | --- | --- |
|  | **N** | **Median** | **Mean** | **rSD** | **SD** | **N** | **Median** | **Mean** | **rSD** | **SD** | **FC** | **CI** |  |
| Wisconsin A+, control: CU A- Wisconsin | | | | | | | | | | | | | |
| YKL40 | 49 | 216.4 | 239.1 | 96.3 | 99.3 | 284 | 125.2 | 127.6 | 36.6 | 39.0 | 1.73 | [1.52–1.97] | <0.001 |
| sTREM2 | 49 | 9.36 | 9.87 | 2.65 | 3.50 | 284 | 7.28 | 7.48 | 2.13 | 2.13 | 1.29 | [1.18–1.40] | <0.001 |
| GFAP | 49 | 13.6 | 14.7 | 5.16 | 6.75 | 284 | 7.78 | 8.20 | 2.66 | 2.69 | 1.75 | [1.56–1.96] | <0.001 |
| IL-6 | 49 | 4.17 | 5.24 | 1.24 | 4.78 | 284 | 4.00 | 4.88 | 1.54 | 3.76 | 1.04 | [0.95–1.14] | 0.764 |
| NfL | 49 | 195.2 | 229.2 | 117.5 | 129.3 | 284 | 69.6 | 78.5 | 23.9 | 61.6 | 2.81 | [2.36–3.34] | <0.001 |
| Neurogranin | 49 | 1006.0 | 1101.7 | 355.1 | 567.9 | 284 | 682.4 | 728.3 | 253.5 | 283.1 | 1.47 | [1.32–1.64] | <0.001 |
| S100 | 49 | 1.18 | 1.24 | 0.28 | 0.33 | 284 | 1.12 | 1.15 | 0.26 | 0.32 | 1.05 | [0.98–1.13] | 0.056 |
| α-Syn | 49 | 322.3 | 341.0 | 142.2 | 165.3 | 284 | 193.1 | 208.5 | 81.2 | 85.0 | 1.67 | [1.46–1.91] | <0.001 |
| Aβ40 | 49 | 17.1 | 18.5 | 6.73 | 7.56 | 284 | 17.9 | 18.4 | 5.57 | 6.03 | 0.96 | [0.85–1.07] | 0.764 |
| Aβ42 | 49 | 652.3 | 817.6 | 302.1 | 638.5 | 284 | 1620.8 | 1753.0 | 588.6 | 768.6 | 0.40 | [0.35–0.46] | <0.001 |
| pTau | 49 | 35.6 | 38.4 | 15.6 | 19.7 | 284 | 14.6 | 15.5 | 4.96 | 4.99 | 2.43 | [2.14–2.76] | <0.001 |
| tTau | 49 | 351.7 | 384.0 | 138.0 | 180.3 | 284 | 168.1 | 179.7 | 54.9 | 60.8 | 2.09 | [1.86–2.35] | <0.001 |
| Wisconsin A+, control: CU A- ALFA+ | | | | | | | | | | | | | |
| YKL40 | 49 | 216.4 | 239.1 | 96.3 | 99.3 | 218 | 128.7 | 136.3 | 43.3 | 43.3 | 1.68 | [1.47–1.92] | <0.001 |
| sTREM2 | 49 | 9.36 | 9.87 | 2.65 | 3.50 | 218 | 7.49 | 7.74 | 2.02 | 2.13 | 1.25 | [1.15–1.36] | <0.001 |
| GFAP | 49 | 13.6 | 14.7 | 5.16 | 6.75 | 218 | 6.77 | 7.28 | 2.17 | 2.41 | 2.01 | [1.79–2.26] | <0.001 |
| IL-6 | 49 | 4.17 | 5.24 | 1.24 | 4.78 | 218 | 3.63 | 4.00 | 1.09 | 1.83 | 1.15 | [1.05–1.26] | 0.011 |
| NfL | 49 | 195.2 | 229.2 | 117.5 | 129.3 | 218 | 74.1 | 75.3 | 22.6 | 23.2 | 2.64 | [2.22–3.14] | <0.001 |
| Neurogranin | 49 | 1006.0 | 1101.7 | 355.1 | 567.9 | 218 | 705.8 | 761.5 | 256.3 | 294.7 | 1.43 | [1.28–1.59] | <0.001 |
| S100 | 49 | 1.18 | 1.24 | 0.28 | 0.33 | 218 | 0.96 | 0.99 | 0.21 | 0.21 | 1.23 | [1.15–1.32] | <0.001 |
| α-Syn | 49 | 322.3 | 341.0 | 142.2 | 165.3 | 218 | 187.7 | 234.8 | 81.0 | 222.4 | 1.72 | [1.50–1.97] | <0.001 |
| Aβ40 | 49 | 17.1 | 18.5 | 6.73 | 7.56 | 218 | 16.8 | 17.5 | 5.27 | 5.10 | 1.02 | [0.90–1.14] | 0.830 |
| Aβ42 | 49 | 652.3 | 817.6 | 302.1 | 638.5 | 218 | 1451.0 | 1564.1 | 560.6 | 591.3 | 0.45 | [0.39–0.52] | <0.001 |
| pTau | 49 | 35.6 | 38.4 | 15.6 | 19.7 | 204 | 14.0 | 15.0 | 4.74 | 4.94 | 2.54 | [2.23–2.89] | <0.001 |
| tTau | 49 | 351.7 | 384.0 | 138.0 | 180.3 | 217 | 172.7 | 182.7 | 54.3 | 56.3 | 2.04 | [1.81–2.29] | <0.001 |
| Abby/Blaze all, control: CU A- ALFA+ | | | | | | | | | | | | | |
| YKL40 | 163 | 191.6 | 208.6 | 72.7 | 76.3 | 218 | 128.7 | 136.3 | 43.3 | 43.3 | 1.49 | [1.38-1.60] | <0.001 |
| sTREM2 | 163 | 8.93 | 9.48 | 2.71 | 2.84 | 218 | 7.49 | 7.74 | 2.02 | 2.13 | 1.19 | [1.12-1.27] | <0.001 |
| GFAP | 164 | 10.6 | 11.8 | 4.57 | 5.00 | 218 | 6.77 | 7.28 | 2.17 | 2.41 | 1.56 | [1.44-1.69] | <0.001 |
| IL-6 | 163 | 3.08 | 3.41 | 1.09 | 2.44 | 218 | 3.63 | 4.00 | 1.09 | 1.83 | 0.85 | [0.79-0.91] | <0.001 |
| NfL | 164 | 189.6 | 218.6 | 69.0 | 174.5 | 218 | 74.1 | 75.3 | 22.6 | 23.2 | 2.56 | [2.39–2.74] | <0.001 |
| Neurogranin | 164 | 999.9 | 1107.1 | 501.0 | 493.9 | 218 | 705.8 | 761.5 | 256.3 | 294.7 | 1.42 | [1.29–1.55] | <0.001 |
| S100 | 164 | 1.10 | 1.14 | 0.25 | 0.31 | 218 | 0.96 | 0.99 | 0.21 | 0.21 | 1.15 | [1.10–1.21] | <0.001 |
| α-Syn | 164 | 379.5 | 483.9 | 200.2 | 480.0 | 218 | 187.7 | 234.8 | 81.0 | 222.4 | 2.02 | [1.83–2.23] | <0.001 |
| Aβ40 | 163 | 15.8 | 16.4 | 5.25 | 5.28 | 218 | 16.8 | 17.5 | 5.27 | 5.10 | 0.94 | [0.88–1.00] | 0.002 |
| Aβ42 | 163 | 576.8 | 634.9 | 190.8 | 237.9 | 218 | 1451.0 | 1564.1 | 560.6 | 591.3 | 0.40 | [0.37–0.43] | <0.001 |
| pTau | 164 | 35.5 | 38.0 | 17.4 | 16.9 | 204 | 14.0 | 15.0 | 4.74 | 4.94 | 2.53 | [2.32–2.76] | <0.001 |
| tTau | 163 | 345.6 | 370.9 | 163.0 | 153.5 | 217 | 172.7 | 182.7 | 54.3 | 56.3 | 2.00 | [1.84–2.18] | <0.001 |
| Abby/Blaze all, control: CU A- Wisconsin | | | | | | | | | | | | | |
| YKL40 | 163 | 191.6 | 208.6 | 72.7 | 76.3 | 284 | 125.2 | 127.6 | 36.6 | 39.0 | 1.53 | [1.43–1.64] | <0.001 |
| sTREM2 | 163 | 8.93 | 9.48 | 2.71 | 2.84 | 284 | 7.28 | 7.48 | 2.13 | 2.13 | 1.23 | [1.16–1.30] | <0.001 |
| GFAP | 164 | 10.6 | 11.8 | 4.57 | 5.00 | 284 | 7.78 | 8.20 | 2.66 | 2.69 | 1.36 | [1.26–1.47] | <0.001 |
| IL-6 | 163 | 3.08 | 3.41 | 1.09 | 2.44 | 284 | 4.00 | 4.88 | 1.54 | 3.76 | 0.77 | [0.72–0.83] | <0.001 |
| NfL | 164 | 189.6 | 218.6 | 69.0 | 174.5 | 284 | 69.6 | 78.5 | 23.9 | 61.6 | 2.73 | [2.55–2.92] | <0.001 |
| Neurogranin | 164 | 999.9 | 1107.1 | 500.9 | 493.9 | 284 | 682.4 | 728.3 | 253.5 | 283.1 | 1.47 | [1.34–1.60] | <0.001 |
| S100 | 164 | 1.10 | 1.14 | 0.25 | 0.31 | 284 | 1.12 | 1.15 | 0.26 | 0.32 | 0.99 | [0.94–1.03] | 0.455 |
| α-Syn | 164 | 379.5 | 483.9 | 200.1 | 480.0 | 284 | 193.1 | 208.5 | 81.2 | 85.0 | 1.97 | [1.79–2.16] | <0.001 |
| Aβ40 | 163 | 15.8 | 16.4 | 5.25 | 5.28 | 284 | 17.9 | 18.4 | 5.57 | 6.03 | 0.88 | [0.83–0.94] | 0.001 |
| Aβ42 | 163 | 576.8 | 634.9 | 190.8 | 237.9 | 284 | 1620.8 | 1753.0 | 588.6 | 768.6 | 0.36 | [0.33–0.38] | <0.001 |
| pTau | 164 | 35.5 | 38.0 | 17.4 | 16.9 | 284 | 14.6 | 15.5 | 4.96 | 4.99 | 2.42 | [2.23–2.64] | <0.001 |
| tTau | 163 | 345.6 | 370.9 | 163.0 | 153.5 | 284 | 168.1 | 179.7 | 54.9 | 60.8 | 2.06 | [1.90–2.23] | <0.001 |

p-values are adjusted using FDR method for each cohort, amyloid-β status, and disease stage comparison independently.

*Abbreviations*: Aβ42, amyloid-β_1–42_; Aβ40, amyloid-β_1–40_; α-Syn, alpha-synuclein; CU, cognitively unimpaired; FDR, False Discovery Rate; GFAP, glial fibrillary acidic protein; IL, interleukin; NfL, neurofilament light; pTau, phosphorylated tau; rSD, robust standard deviation; SD, standard deviation; sTREM2, soluble triggering receptor expressed on myeloid cells 2; tTau, total tau; YKL40, chitinase-3-like protein-1

**Table S11** AUC and 95% confidence interval for the ROC curve by disease stage versus CU A- individuals aged <65 years (amyloid status as defined by Aβ42/Aβ40)

|  | **ALFA CU A+** | | **Wisconsin CU A+** | | **Wisconsin MCI A+** | | **Wisconsin AD-dementia** | |
| --- | --- | --- | --- | --- | --- | --- | --- | --- |
| Biomarker | AUC | 95% CI | AUC | 95% CI | AUC | 95% CI | AUC | 95% CI |
| YKL40 | 60.4 | 54.1–66.6 | 62.1 | 56.6–67.6 | 87.2 | 79.9–94.5 | 88.7 | 83.7–93.6 |
| sTREM2 | 53.1 | 46.8–59.4 | 57.0 | 51.4–62.6 | 74.6 | 64.7–84.5 | 71.6 | 63.0–80.2 |
| GFAP | 60.9 | 54.7–67.0 | 61.3 | 55.9–66.8 | 92.5 | 88.0–97.1 | 87.1 | 81.7–92.4 |
| IL-6 | 50.0 | 43.7–56.3 | 54.7 | 49.2–60.3 | 62.8 | 53.0–72.6 | 51.6 | 43.2–60.1 |
| NfL | 65.3 | 59.4–71.2 | 69.4 | 64.2–74.5 | 95.6 | 93.3–97.9 | 96.9 | 95.2–98.7 |
| Neurogranin | 60.2 | 54.1–66.3 | 63.3 | 57.7–68.9 | 73.1 | 63.1–83.1 | 76.4 | 69.1–83.8 |
| S100 | 57.9 | 51.5–64.2 | 50.8 | 45.1–56.4 | 66.3 | 55.9–76.8 | 58.9 | 49.7–68.0 |
| α-Syn | 53.0 | 46.9–59.2 | 60.5 | 55.0–66.0 | 78.6 | 70.2–87.0 | 77.2 | 69.4–85.1 |
| Aβ40 | 49.4 | 43.2–55.6 | 48.3 | 42.6–54.1 | 58.7 | 47.7–69.7 | 51.4 | 42.0–60.8 |
| Aβ42 | 86.5 | 82.7–90.3 | 82.5 | 78.5–86.5 | 92.3 | 86.8–97.8 | 90.6 | 84.6–96.7 |
| pTau | 65.8 | 59.8–71.8 | 69.3 | 63.9–74.8 | 91.3 | 85.9–96.7 | 92.8 | 88.3–97.3 |
| tTau | 66.0 | 60.1–71.9 | 69.0 | 63.6–74.3 | 88.9 | 82.6–95.2 | 92.1 | 87.8–96.4 |

*Abbreviations*: Aβ42, amyloid-β_1–42_; Aβ40, amyloid-β_1–40_; α-Syn, alpha-synuclein; AD, Alzheimer’s disease; AUC, area under the curve; CU, cognitively unimpaired; GFAP, glial fibrillary acidic protein; IL, interleukin; MCI, mild cognitive impairment; NfL, neurofilament light; pTau, phosphorylated tau; ROC, receiver operating characteristics; sTREM2, soluble triggering receptor expressed on myeloid cells 2; tTau, total tau; YKL40, chitinase-3-like protein-1

**Fig. S1** Biomarker distributions for uncorrected and corrected (A) Aβ42, (B) Aβ40, and (C) α-Syn in the reference group (i.e. CU individuals who were *APOE-ε4* non-carriers and aged <65 years). Aβ40, amyloid-β_1–40_; Aβ42, amyloid-β_1–42_; α-Syn, alpha-synuclein; CU, cognitively unimpaired


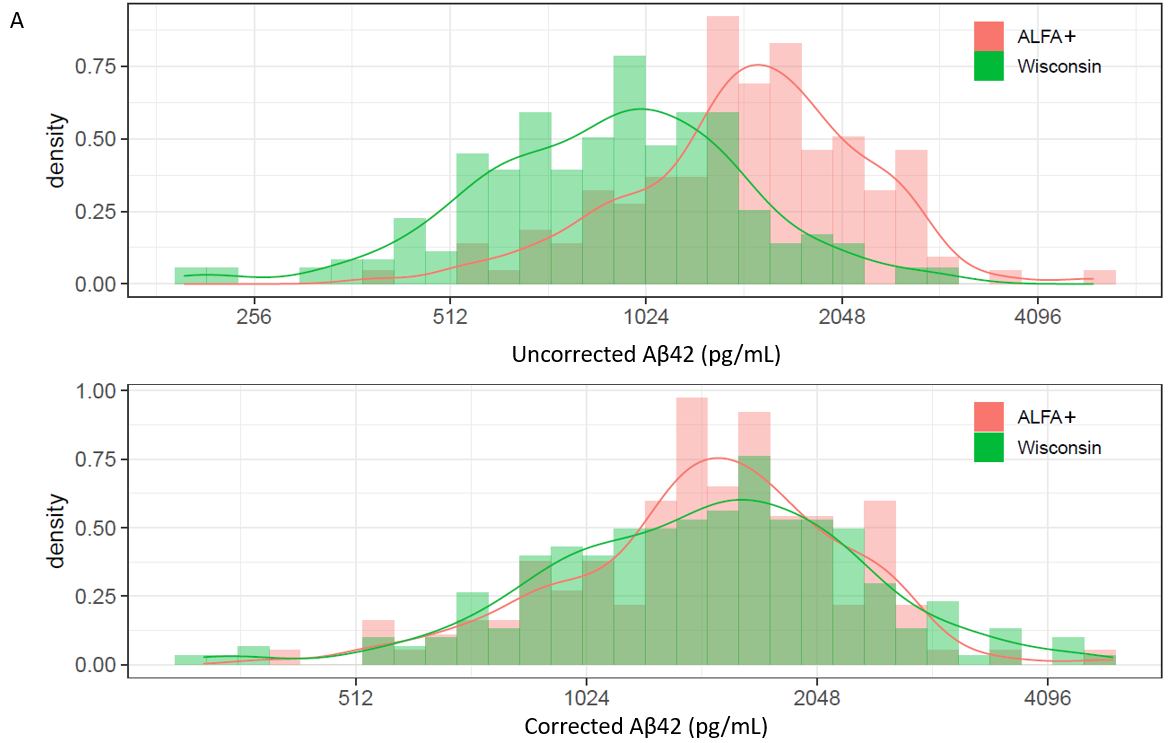


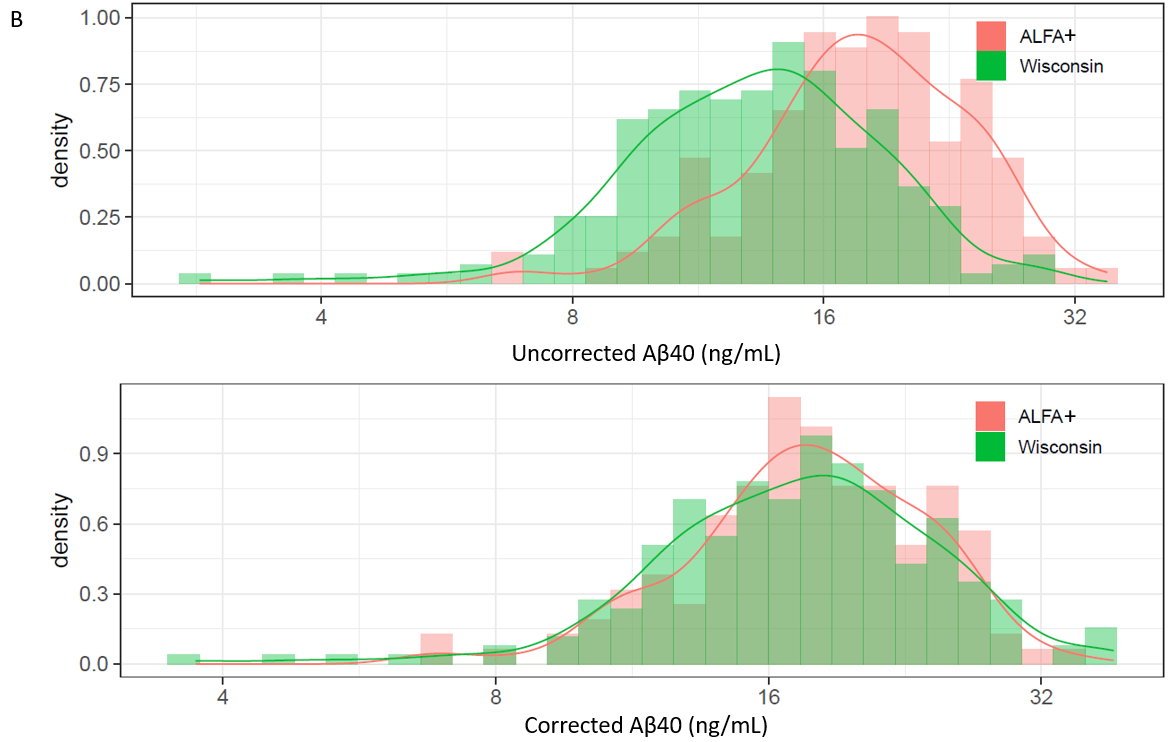


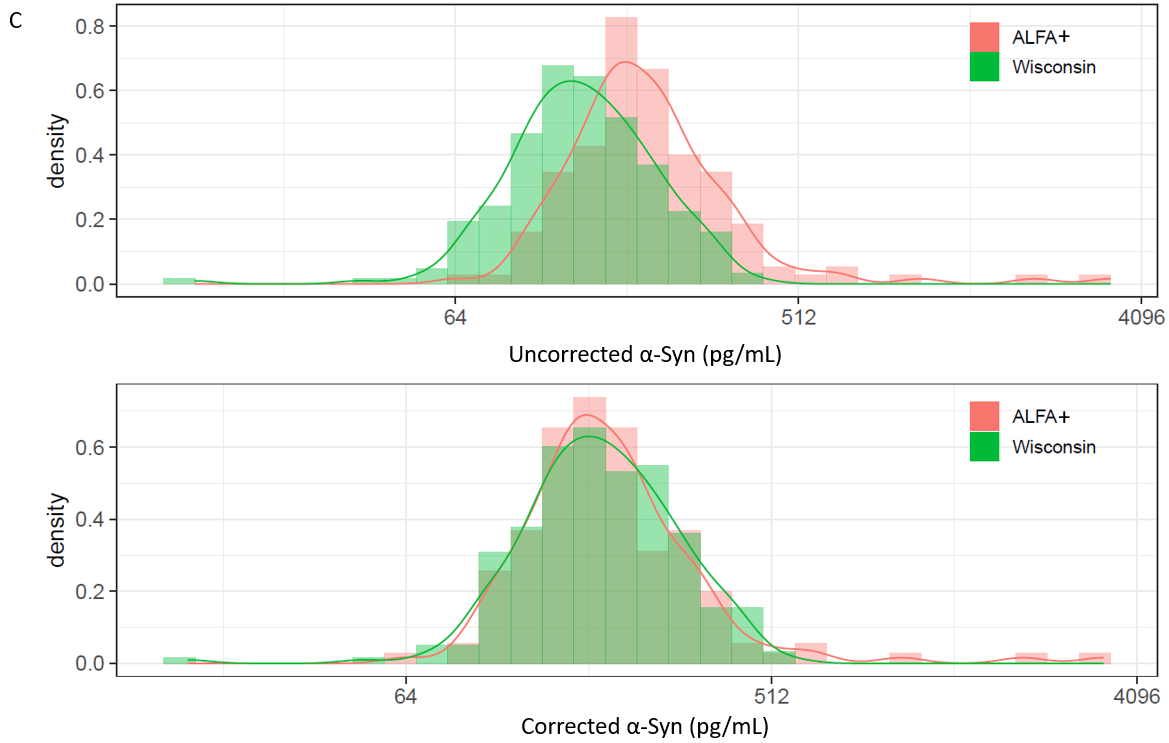


**Fig. S2** Baseline correlation between NTK biomarkers in all CU cases ([A] ALFA+ cohort and [B] Wisconsin cohort) and baseline correlation between NTK biomarkers and all AD-dementia cases ([C] Wisconsin cohort and [D] Abby/Blaze cohort). White boxes represent biomarker concentration comparisons that are not significant (*p*>0.05). Aβ40, amyloid-β_1–40_; Aβ42, amyloid-β_1–42_; AD, Alzheimer’s disease; BL, baseline; CU, cognitively unimpaired; GFAP, glial fibrillary acidic protein; IL, interleukin; NfL, neurofilament light; NTK, NeuroToolKit; pTau, phosphorylated tau; sTREM2, soluble triggering receptor expressed on myeloid cells 2; tTau, total tau; YKL40, chitinase-3-like protein-1
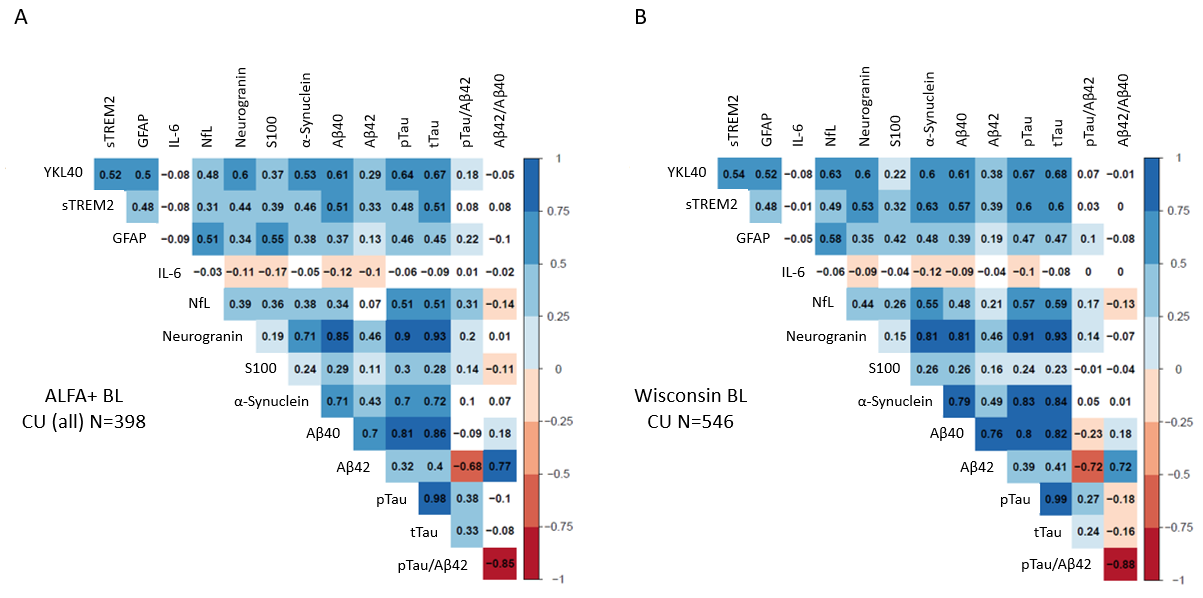


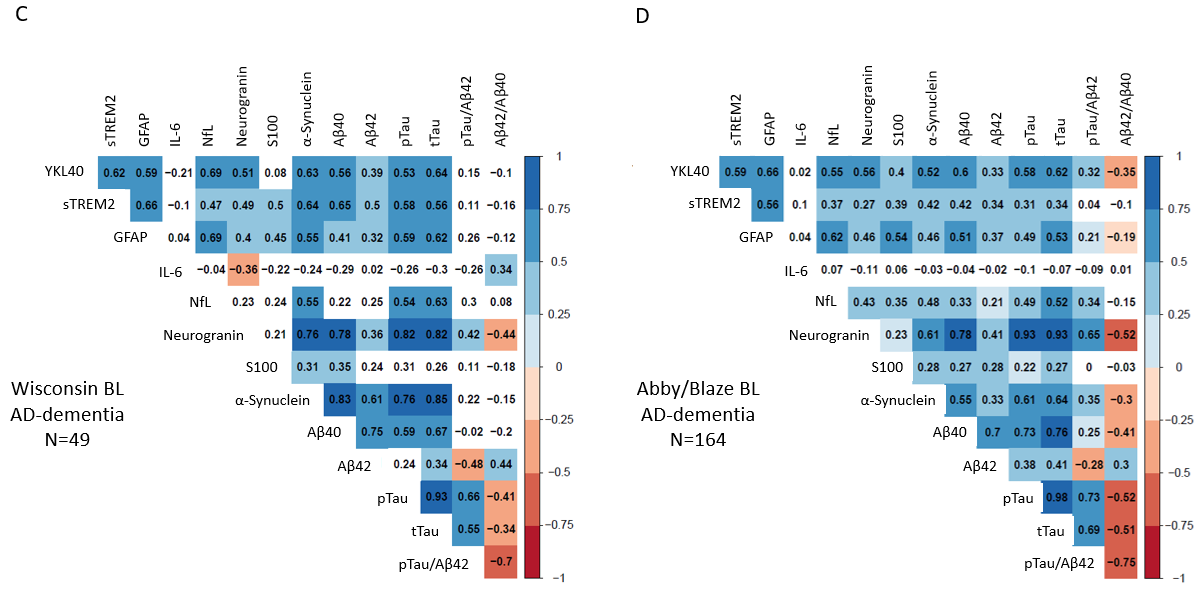


**Fig. S3** ROC analysis of CU A- individuals compared with CU A+ individuals, patients with MCI A+, and patients with AD-dementia (amyloid status as defined by Aβ42/Aβ40). Aβ42, amyloid-β_1–42_; Aβ40, amyloid-β_1–40_; AD, Alzheimer’s disease; CU, cognitively unimpaired; GFAP, glial fibrillary acidic protein; IL, interleukin; MCI, mild cognitive impairment; NfL, neurofilament light; pTau, phosphorylated tau; ROC, receiver operating characteristics; sTREM2, soluble triggering receptor expressed on myeloid cells 2; tTau, total tau


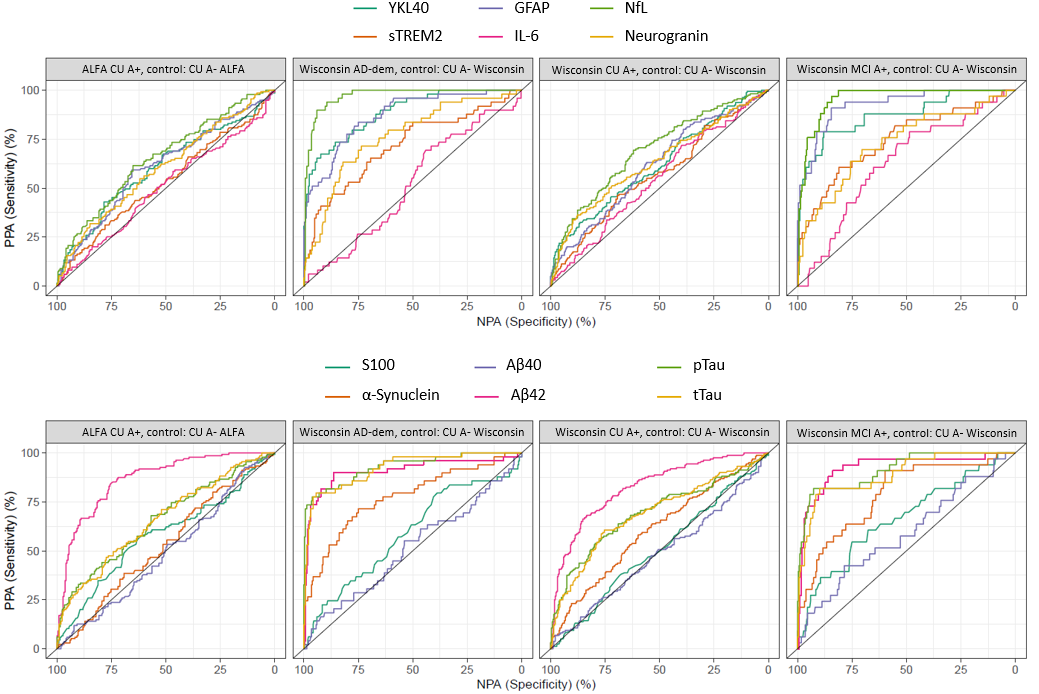


**REFERENCES**

1. Molinuevo, J.L., N. Gramunt, J.D. Gispert, et al. The ALFA project: A research platform to identify early pathophysiological features of Alzheimer's disease. Alzheimers Dement (N Y). 2016;**2**:82-92.

2. Folstein, M.F., S.E. Folstein, and P.R. McHugh. "Mini-mental state". A practical method for grading the cognitive state of patients for the clinician. J Psychiatr Res. 1975;**12**:189-98.

3. Blesa, R., M. Pujol, M. Aguilar, et al. Clinical validity of the 'mini-mental state' for Spanish speaking communities. Neuropsychologia. 2001;**39**:1150-7.

4. Buschke, H., G. Kuslansky, M. Katz, et al. Screening for dementia with the memory impairment screen. Neurology. 1999;**52**:231-8.

5. Bohm, P., J. Pena-Casanova, N. Gramunt, et al. [Spanish version of the Memory Impairment Screen (MIS): normative data and discriminant validity]. Neurologia. 2005;**20**:402-11.

6. Ramier, A.M. and H. Hecaen. [Respective roles of frontal lesions and lesion lateralization in "verbal fluency" deficiencies]. Rev Neurol (Paris). 1970;**123**:17-22.

7. Pena-Casanova, J., S. Quinones-Ubeda, N. Gramunt-Fombuena, et al. Spanish Multicenter Normative Studies (NEURONORMA Project): norms for verbal fluency tests. Arch Clin Neuropsychol. 2009;**24**:395-411.

8. Morris, J.C. The Clinical Dementia Rating (CDR): current version and scoring rules. Neurology. 1993;**43**:2412-4.

9. Goldberg, D., K. Bridges, P. Duncan-Jones, and D. Grayson. Detecting anxiety and depression in general medical settings. BMJ. 1988;**297**:897-9.

10. Monton, C., M.J. Perez Echeverria, R. Campos, J. Garcia Campayo, and A. Lobo. [Anxiety scales and Goldberg's depression: an efficient interview guide for the detection of psychologic distress]. Aten Primaria. 1993;**12**:345-9.

11. Mila-Aloma, M., G. Salvado, J.D. Gispert, et al. Amyloid beta, tau, synaptic, neurodegeneration, and glial biomarkers in the preclinical stage of the Alzheimer's continuum. Alzheimers Dement. 2020;**16**:1358-71.

12. Teunissen, C.E., H. Tumani, S. Engelborghs, and B. Mollenhauer. Biobanking of CSF: international standardization to optimize biomarker development. Clin Biochem. 2014;**47**:288-92.

13. Van Hulle, C., E.M. Jonaitis, T.J. Betthauser, et al. An examination of a novel multipanel of CSF biomarkers in the Alzheimer's disease clinical and pathological continuum. Alzheimers Dement. 2021;**17**:431-45.

14. Cummings, J.L., S. Cohen, C.H. van Dyck, et al. ABBY: A phase 2 randomized trial of crenezumab in mild to moderate Alzheimer disease. Neurology. 2018;**90**:e1889-e97.

15. Salloway, S., L.A. Honigberg, W. Cho, et al. Amyloid positron emission tomography and cerebrospinal fluid results from a crenezumab anti-amyloid-beta antibody double-blind, placebo-controlled, randomized phase II study in mild-to-moderate Alzheimer's disease (BLAZE). Alzheimers Res Ther. 2018;**10**:96.

16. McKhann, G., D. Drachman, M. Folstein, et al. Clinical diagnosis of Alzheimer's disease: report of the NINCDS-ADRDA Work Group under the auspices of Department of Health and Human Services Task Force on Alzheimer's Disease. Neurology. 1984;**34**:939-44.

17. Sheikh, R. and J. Yesavage. Geriatric Depression Scale (GDS): recent evidence and development of a shorter version. Clin Gerontol. 1986;**5**:165–73.

18. O'Bryant, S.E., S.C. Waring, C.M. Cullum, et al. Staging dementia using Clinical Dementia Rating Scale Sum of Boxes scores: a Texas Alzheimer's research consortium study. Arch Neurol. 2008;**65**:1091-5.

19. Berg, L., J.P. Miller, J. Baty, et al. Mild senile dementia of the Alzheimer type. 4. Evaluation of intervention. Ann Neurol. 1992;**31**:242-9.

20. Sano, M., R. Raman, J. Emond, et al. Adding delayed recall to the Alzheimer Disease Assessment Scale is useful in studies of mild cognitive impairment but not Alzheimer disease. Alzheimer Dis Assoc Disord. 2011;**25**:122-7.
